# The First Transcriptome Assembly of Yenyuan Stream Salamander (*Batrachuperus yenyuanensis*) Provides Novel Insights into Its Molecular Evolution

**DOI:** 10.3390/ijms20071529

**Published:** 2019-03-27

**Authors:** Jianli Xiong, Yunyun Lv, Yong Huang, Qiangqiang Liu

**Affiliations:** 1 Laboratory of Adaptation and Evolution of Aquatic Animals, College of Animal Science and Technology, Henan University of Science and Technology, Luoyang 471023, China; huangyong@haust.edu.cn (Y.H.); liuqiang_1017@sina.com (Q.L.); 2 BGI Education Center, University of Chinese Academy of Sciences, Shenzhen 518083, China; lvyunyun@genomics.com

**Keywords:** salamander, transcriptome, regeneration, divergence

## Abstract

The Yenyuan stream salamander (*Batrachuperus yenyuanensis*) has been previously evaluated with regards to phylogeny, population genetics, and hematology, but genomic information is sparse due to the giant genome size of salamanders which contain highly repetitive sequences, thus resulting in the lack of a complete reference genome. This study evaluates the encoding genetic sequences and provides the first transcriptome assembly of Yenyuan stream salamander based on mixed samples from the liver, spermary, muscle and spleen tissues. Using this transcriptome assembly and available encoding sequences from other vertebrates, the gene families, phylogenetic status, and species divergence time were compared or estimated. A total of 13,750 encoding sequences were successfully obtained from the transcriptome assembly of Yenyuan stream salamander, estimated to contain 40.1% of the unigenes represented in tetrapod databases. A total of 88.79% of these genes could be annotated to a biological function by current databases. Through gene family clustering, we found multiple possible isoforms of the *Scribble* gene—whose function is related to regeneration—based on sequence similarity. Meanwhile, we constructed a robust phylogenetic tree based on 56 single-copy orthologues, which indicates that based on phylogenetic position, the Yenyuan stream salamander presents the closest relationship with the Chinese giant salamander (*Andrias davidianus*) of the investigated vertebrates. Based on the fossil-calibrated phylogeny, we estimated that the lineage divergence between the ancestral Yenyuan stream salamander and the Chinese giant salamander may have occurred during the Cretaceous period (~78.4 million years ago). In conclusion, this study not only provides a candidate gene that is valuable for exploring the remarkable capacity of regeneration in the future, but also gives an interesting insight into the understanding of Yenyuan stream salamander by this first transcriptome assembly.

## 1. Introduction

The Yenyuan stream salamander (*Batrachuperus yenyuanensis* [1]), belonging to the family Hynobiidae, order Caudata, is an aquatic organism that is endemic to Western China. This species has a low migration capacity and resides in the eastern edge around the Tibetan Plateau at altitudes ranging from 2900 to 4400 m [2]. Only a few studies have paid attention to this species and contributed to the limited understanding of its phylogeny and population genetics [3,4]. It was revealed that past variance events might have resulted in a dominant effect on their evolution [4]. The elevation, topography, and cold tolerance may have driven the evolutionary patterns of diversification and demography in this species and other relatives [5]. In addition, a recent hematological study of hynodiid salamanders indicated that hematological parameters presented a species-specific and genus-specific disparity, which may be related to adaption to the corresponding living environment [6].

Besides these studies, genomic studies have given no attention to this species and only a little to other salamanders [7]. The reason is that salamanders have highly repetitive sequences in their genomes, leading to an extremely large genome size. The only complete genomic assembly of a salamander (Mexican axolotl, *Ambystoma mexicanum*) contains 30 gigabases (Gb) of content [7]. The highly repetitive sequences bring about a technical obstacle for complete genome assembly, despite a third-generation sequencing platform which seems to have overcome this obstacle to a certain extent. However, large amounts of high-throughput third-generation sequencing would be expensive for such a large genome size. Although these reasons hinder the completion of whole-genome assembly for salamanders, the corresponding whole protein-encoding sequences present a similar number to other vertebrates. An example would be the Mexican axolotl [7], which has more than twenty thousand protein-coding genes, which is similar to other vertebrates. Thus, an alternative approach to understanding the genomic information of salamander might be available in the transcriptome, which contains an amount of accessible encoding sequences by RNA sequencing (RNA-seq). This strategy provides a direct way of understanding the encoding genetic sequences in salamander.

In this study, we sampled Yenyuan stream salamanders in southwestern China. Considering the specificity of RNA expression in different tissues, we collected multiple tissues to generate a mixed transcriptome library, which can contain more encoding genetic sequences than just one type of tissue. The mixed samples contained the liver, spermary, muscle, and spleen which are crucial for metabolism, reproduction, growth, and immunity. Through RNA-seq, we successfully assembled the first transcriptome of Yenyuan stream salamander. Subsequently, we utilized this assembly to compare the inside gene families with other vertebrates, and deduced the phylogenetic position and lineage divergence time between this species and other vertebrates. Using this approach, we attempted to provide an improved recognition of the molecular evolution of Yenyuan stream salamander.

## 2. Results

### 2.1. Sampling and Transcriptome Assembly of the Yenyuan Stream Salamander

**Figure 1:**
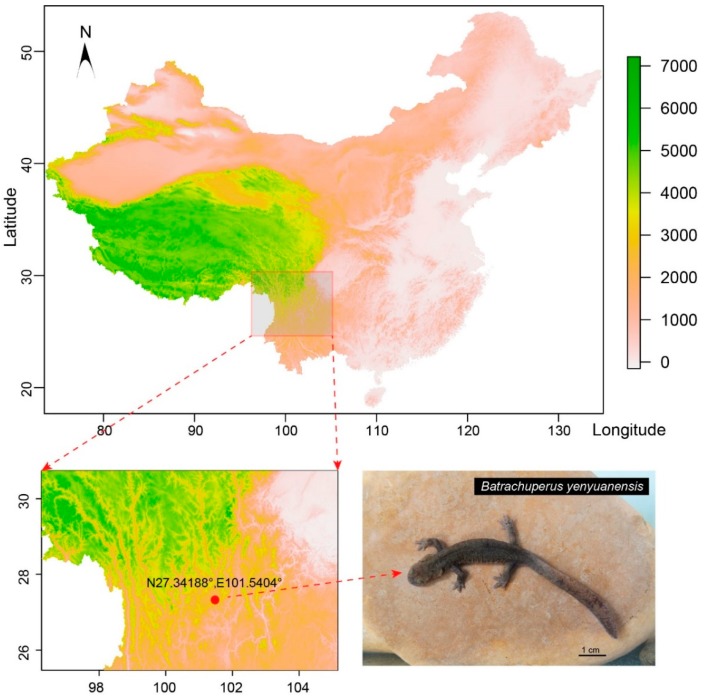
The sampling location and a sample picture of Yenyuan stream salamander. The red dotted line(s) in left indicate the amplified sampling location and right indicates the sampling salamander.

The Yenyuan stream salamanders (*B. yenyuanensis*) used in this study were sampled from Bailing Mountain, Yanyuan Town, Xichang City, Sichuan Province, China (elevation: 3846 m; see details in Figure 1). An adult individual, with a snout–vent length (SVL) >8 cm, was chosen for the study. Its sex was determined to be male by anatomy. Sample tissues from the liver, spermary, muscle, and spleen were collected to generate a mixed sample. The tissues were initially placed in a 1.5 mL tube filled with RNAlater (Ambion, Carlsbad, CA, USA). The tube was then immediately frozen in liquid nitrogen for storage. This sampled individual was also vouchered in Henan University of Science and Technology Museum and numbered as “HNUSTM201605092”. All salamander handling and experimental procedures performed in this study were approved (January 1, 2015) by the Animal Care and Use Committee of the College of Animal Science and Technology, Henan University of Science and Technology (CAST2015040010).

After the completion of transcriptome sequencing and removal of low-quality reads, 24,962,019 clean reads containing 3,744,302,850 bases were retained. A total of 115,495 raw contigs were initially assembled with the whole content of 88,019,795 bp (see the length distribution in Figure S1). After removing possible redundant contigs, the retained unigenes contained a total length of 71,498,614 bp from 70,540 sequences (see the length distribution in Figure S2). Based on the expression calculation of fragments per kilobase million (FPKM), we screened 26,179 unigenes with an FPKM expression level ≥1 as relatively reliable unigenes. From these unigenes, we predicted 13,750 protein-coding sequences (see the length distribution in Figure S3), which presented 40.1% completeness (see details in Table S1) by searching 3950 BUSCO groups from tetrapods. Overall, in the functional annotations, 88.79% of the predicated protein-coding sequences were annotated as biological function (see annotated states in Table S2). Based on the expression of FPKM, we determined the biological function of those genes included in the list of top 20 expression levels, of which 18 genes were able to be allocated to a biological function. Two of them may not be included in the database. The gene with the highest expressional level (FPKM = 15,004) was *eEF1a1*, which encodes the protein elongation factor 1-alpha 1, which is responsible for the enzymatic delivery of aminoacyl tRNAs to the ribosome. Details of the 18 annotated genes with the highest expression are listed in Table S3.

### 2.2. Gene Families of the Yenyuan Stream Salamander

**Figure 2:**
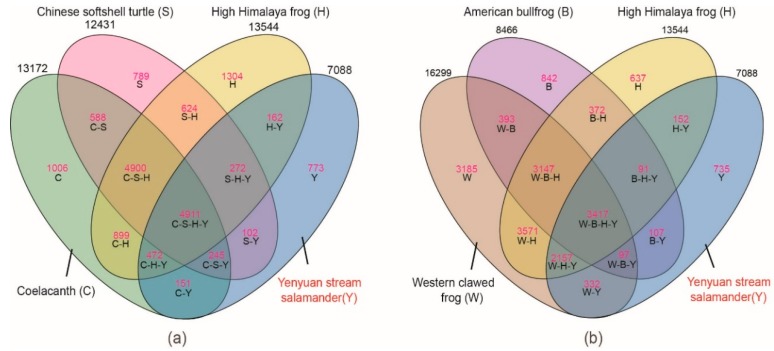
Gene family clustering among the six vertebrates: (**a**) The clustering state among coelacanth, Chinese softshell turtle, High Himalaya frog, and Yenyuan stream salamander; (**b**) The clustering state among Western clawed frog, American bullfrog, High Himalaya frog, and Yenyuan stream salamander.

By comparing the gene families of Yenyuan stream salamander and other vertebrates, we found 10,829 sequences from Yenyuan stream salamander that can be clustered with other species and distributed in 7088 gene families (Table S4). There are 4911 gene families shared among the coelacanth, Chinese softshell turtle, High Himalaya frog, and Yenyuan stream salamander. There are 162 special gene families that are shared between the Himalaya frog and Yenyuan stream salamander, which is higher than the coelacanth (151) and Chinese softshell turtles (102) (Figure 2a). We also found 3417 gene families shared by four amphibians, including Western clawed frog, American bullfrog, High Himalaya frog, and Yenyuan stream salamander. The Western clawed frog has 332 special gene families shared with the Yenyuan stream salamander, which is higher than the Himalaya frog (152) and American bullfrog (107) (Figure 2b). These results indicate that the Yenyuan stream salamander presents the highest number of shared gene families with the Western clawed frog.

In addition, through gene family clustering, we found nine *Scribble* isoforms in the transcriptome of the Yenyuan stream salamander (Figure S4). These isoforms displayed multiple alternative splicing forms (see Figure S4). The *Scribble* isoform encoding the scribbled planar cell polarity protein is a member of the Hippo pathway, which plays a crucial role in animal development and regeneration [8]. These *Scribble* isoforms in Yenyuan stream salamander may be responsible for its remarkable regeneration capacity. Thus, we provide a candidate gene which deserves further exploration regarding its role in regeneration in salamanders.

### 2.3. Evolutionary Status of the Yenyuan Stream Salamander

**Figure 3:**
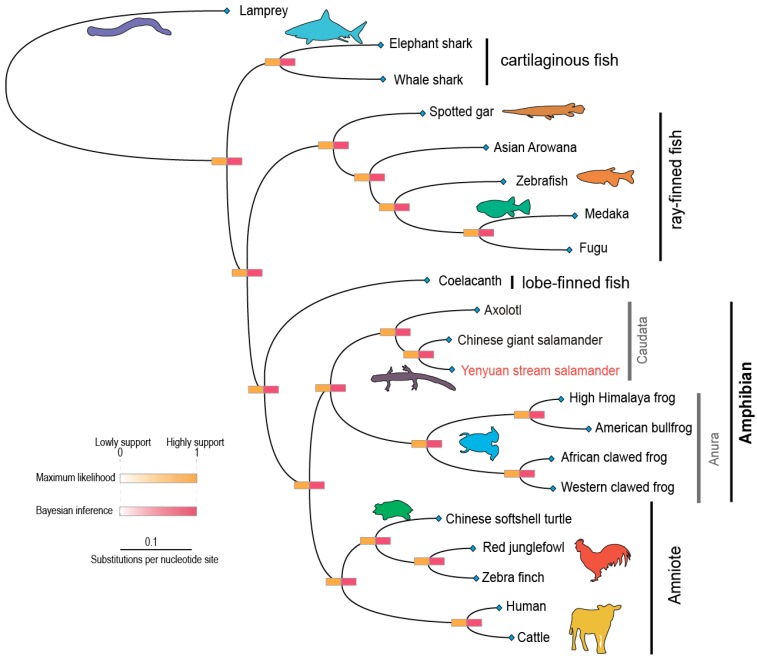
Phylogenetic construction of the Yenyuan stream salamander and other vertebrates by 56 single-copy orthologues based on maximum likelihood (ML) and Bayesian inference (BI).

**Figure 4:**
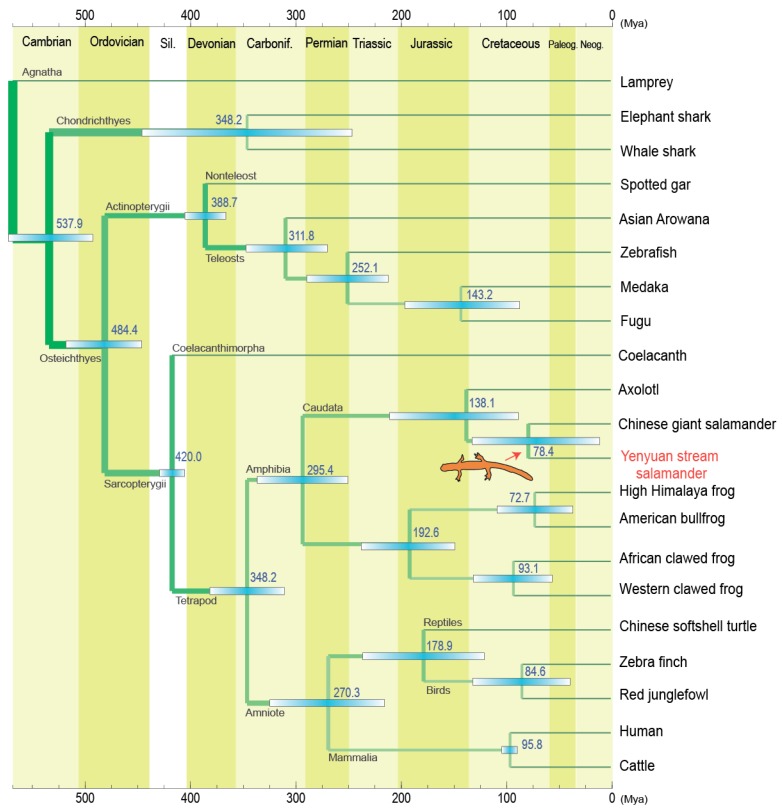
Molecular timing of species divergences among the Yenyuan stream salamander and other investigated vertebrates. The bar within each node represents the confidence interval (5~95%) for time divergence.

A total of 56 single-copy gene families are shared between the Yenyuan stream salamander and other vertebrates. The total length of these single-copy orthologues reached 127,746 bp, and all the first positions from each codon were cascaded with a length of 42,582 bp. Based on this dataset, we constructed a robust evolutionary relationship between the Yenyuan stream salamander and 20 other species. The topologies deduced from maximum likelihood (ML) and Bayesian inference (BI) were totally coincided; both presented strong branch supports (Figure 3). In these robust topologies, we found that the Yenyuan stream salamander originated from a second primitive status in the tetrapod, which descended from the coelacanth; its ancestral branch formed a sister relationship with the ancestral branch of Anura. This relationship coincides with the general recognition that Caudata and Anura originated from the same lineage. In Caudata, the Yenyuan stream salamander presents a closer relationship with the Chinese giant salamander (*Andrias davidianus*) than with the axolotl (*A. mexicanum*). Based on the fossil-calibrated phylogeny, the ancestral lineage divergence between the Yenyuan stream salamander and the Chinese giant salamander was estimated at Cretaceous (~78.4 Mya) and the split between Caudata and Anura occurred at about Carboniferous (~295 Mya) (Figure 4).

## 3. Discussion

In this study, we provided the first transcriptome assembly of Yenyuan stream salamander. This assembly contains 13,750 predicted protein-coding sequences, of which 88.79% were annotated as biological functions. Although the large genome size challenged the whole-genome assembly of salamanders, we were able to provide alternative encoding of the genetic information of Yenyuan stream salamander from multiple tissues using RNA-seq. Previous studies on this species were limited to mitochondrial genes [4,5]. In this current study, we obtained numerous single-copy nuclear genes from Yenyuan stream salamander which contain phylogenic signals through comparison with other salamanders and vertebrates. These nuclear genes may be considered as effective candidate phylogenetic markers that are appropriate for further evolutionary studies.

Coelacanths are known as the slowest-evolving vertebrate [9]. We observed that the branch length of Yenyuan stream salamander, representing an accumulated variation in nucleotide substitution, is closer to the length of Chinese giant salamander and coelacanth. It is apparently shorter than frogs such as the High Himalaya frog, American bullfrog, African clawed frog, and Western clawed frog. In addition, through gene family clustering, we observed that more gene families were shared with coelacanth than with Chinese softshell turtle. These results delineate a conserved evolution of the Yenyuan stream salamander which is similar to the Chinese giant salamander and the coelacanth.

Previous transcriptome studies on salamanders gave insights into the identification of the sex-biased genes in Chinese giant salamander [10], but more transcriptome studies paid attention to the exploration of the genes related to the regenerative capacities of salamanders such as axolotl (*A. mexicanum*) and two of its relatives (*A. andersoni* and *A. maculatum*) [11], Eastern newt (*Notophthalmus viridescens*) [12], Japanese fire belly newt (*Cynops pyrrhogaster*) [13], plethodontid salamander (*Bolitoglossa ramosi*) [14], and Iberian ribbed newt (*Pleurodeles waltl*) [15]. These salamanders were investigated due to having remarkable regenerative capabilities in the retina, lens, heart, appendage, tail, and limb [10,11,12,13,14,15,16]. It has been found that the species-restricted genes may contribute to limb regeneration [7]. In this study, we found nine *Scribble* isoforms in the transcriptome assembly of Yenyuan stream salamander. This encoding scribbled planar cell polarity protein is involved in the Hippo tumor-suppressor pathway, which plays a crucial role in animal development and regeneration [8]. A previous study demonstrated that this pathway is required for *Xenopus* limb bud regeneration [17]. In addition, the protein encoded by *Scribble* is required in central nervous system (CNS) myelination and remyelination [18]. Based on these roles of the *Scribble*-encoded protein, we speculated that multiple *Scribble* isoforms may be involved in the remarkable regenerative capability of the salamander. Thus, we provided a valuable candidate gene for the study of regeneration in salamander, but it needs to be verified further.

In addition, the timing of lineage divergence between Caudata and Anura had been estimated by many studies [19,20,21,22,23,24]. However, controversies have also been presented in previous studies. The reason behind these controversies is the presence of differences in the evolutionary rates within different phylogenetic markers and the use of divergent fossil-calibrated points. Based on the 56 single-copy orthologues and fossil-calibrated phylogenetic topology, we estimated that the split between Caudata and Anura occurred at about 295.4 Mya, which is close to the previous estimates of 302.5 [21], 305.7 [19], and 308 Mya [24]. Our current study was based on numerous nuclear genes, thus giving new support to these estimates.

## 4. Materials and Methods

### 4.1. Total RNA Extraction and Sequencing

Total RNA was extracted according to the protocol of PureLink™ RNA Mini Kit (Thermo Scientific, Waltham, MA, USA). The transcriptome isolation started with phase separation using TRIzol reagent (Ambion, Carlsbad, CA, USA), followed by binding, washing, and elution. The completeness and quality of the RNA was examined by 1.2% agarose electrophoresis and NanoDrop 2000 Nucleic Acid Protein Detector (Thermo Scientific, Wilmington, NC, USA). The RNA integrity number (RIN) was examined using an Agilent 2100 Bioanalyzer (Agilent, Santa Clara, CA, USA) with Agilent RNA 6000 Nano Reagents (Agilent, Santa Clara, CA, USA). Samples with RIN ≥8.0 were chosen for next generation sequencing (NGS). The RNA sample was separated and purified using oligo(dT) magnetic beads and a magnetic separator (Illumina, San Diego, CA, USA). It was subsequently broken into fragments to construct a cDNA library by PCR amplification and enrichment. This cDNA library was used to generate transcriptome (RNA-seq) data on Illumina HiSeq 4000 platform (San Diego, CA, USA) with paired-end reads at a length of 150 bp.

### 4.2. De Novo Transcriptome Assembly and Annotation

Prior to the de novo transcriptome assembly, we carried out a filtering process to remove the redundant reads. The reads with adaptor contamination were discarded first. The low-quality reads with ambiguous characters “N” were also discarded. Finally, the reads with more than 10% Q < 20 bases were ruled out. A de novo assembly was performed using Trinity (version 2.0.6, [25]) with the minimum assembled contig length of 150 bp and maximum length expected between fragment pairs of 530 bp; reads outside this distance were treated as single-end. After the initial assembly, we performed contig clustering to obtain the dataset of all unigenes, which was implemented in the TGICL (version 2.0.6, [26]) program. All reads were aligned to the dataset of unigenes using Bowtie 2 (version 2.2.5, [27]) and, subsequently, the expression level of each unigene calculated using RSEM (version 1.2.12, [28]). In this step, the unigenes with an FPKM value ≥ 1 were regarded as reliable unigenes, and were used for further analysis. We employed BUSCOs [29] to assess the completeness of the unigenes. All reliable unigenes were used to predict the encoding sequences by Transdecoder (version 3.0.1, [25]) with a minimum length of more than 150 bp. To understand the biological function of the predicted encoding sequences, we annotated them using information from five databases, including Nr (non-redundant) [30], Swiss-Prot [31], KEGG (Kyoto Encyclopedia of Genes and Genomes) [32], and KOG (EuKaryotic Orthologous Groups) [33] databases.

### 4.3. Gene Family Comparisons

To determine the phylogenetic position of Yenyuan stream salamander in vertebrates, we chose whole-genome encoding sequences from 17 vertebrates to perform the phylogenetic analysis. The jawless vertebrate known as sea lamprey was employed as the outgroup, and all the encoding sequences from Yenyuan stream salamander and 17 other vertebrates were subjected as in-group species, which contained the lamprey (*Petromyzon marinus*) [34], elephant shark (*Callorhinchus milii*) [35], whale shark (*Rhincodon typus*) [36], spotted gar (*Lepisosteus oculatus*) [37], Asian arowana (*Scleropages formosus*) [38], zebrafish (*Danio rerio*) [39], medaka (*Oryzias latipes*) [40], fugu (*Takifugu rubripes*) [41], coelacanth (*Latimeria chalumnae*) [9], High Himalaya frog (*Nanorana parkeri*) [42], American bullfrog (*Rana catesbeiana*) [43], African clawed frog (*Xenopus laevis*) [44], Western clawed frog (*Xenopus tropicalis*) [45], Chinese softshell turtle (*Pelodiscus sinensis*) [46], zebra finch (*Taeniopygia guttata*) [47], red junglefowl (*Gallus gallus*) [48], human (*Homo sapiens*) [49], and cattle (*Bos taurus*) [50]. Additionally, to further investigate the phylogenetic position of Yenyuan stream salamander in order Caudata, axolotl and Chinese giant salamander were also added into the phylogenetic analysis. The transcriptome assembly of axolotl and the RNA-seq raw data of Chinese giant salamander were retrieved from the TSA (GFZP00000000.1) and SRA (SRR7396733) databases of NCBI, respectively. The RNA-seq raw data were assembled according to the process used for Yenyuan stream salamander.

Initially, we translated the encoding regions to proteins from each vertebrate and performed all-to-all alignments by BLAST (mode blastp, version 2.26, [51]) with an E-value cutoff of 10^−5^. OrthoMCL (OrthoMCL DB: Ortholog Groups of Protein Sequences, [52]) was employed to distinguish gene families based on the similarity among the all-to-all alignments, and Markov chain clustering (MCL) with the parameter “−inflation 1.5” was assigned. Six species, including African clawed frog, American bullfrog, High Himalaya frog, Western clawed frog, Chinese softshell turtle, and coelacanth were used for gene set comparisons with Yenyuan stream salamander.

### 4.4. Phylogenetic Construction and Estimation of Species Divergence Time

The single-copy orthologues among the investigated species were translated to proteins, and we performed multiple alignments using MUSCLE (version 3.7, [53]). Subsequently, the alignments were converted to corresponding coding sequences (CDS). The first site in each codon was chosen to perform a phylogenetic construction based on the maximum likelihood (ML) method, which was implemented in PhyML (version 3.0, [54]) with a gamma distribution across aligned sites and HKY85 substitution model. The approximate likelihood ratio test (aLRT) was employed to evaluate the branch supports. To further confirm the deduced topology based on ML, we simultaneously performed Bayesian inference (BI) using the software MrBayes (version 3.2.2, [55]) with HKY85 substitution model. Two parallel runs of 200,000 generations and sampling every 200 generations were performed. The initial 25% runs were abandoned due to unreliability, while the retained samples were used to estimate the maximum clade credibility tree.

For estimation of the ancestral split of Yenyuan stream salamander from other vertebrates, we set two fossil-calibrated nodes in the phylogenetic topology to estimate the divergence of the Yenyuan stream salamander from other vertebrates, which was based on the Bayesian method using MCMCtree in the PAML (version 4.9e, [56]). Two fossil-calibrated nodes (C1 and C2) and an additional calibrated node (C3) from a previous study [57] were employed as normal distributions and soft constraint bands (allowing a small probability (0.025) of violation). The C1 calibration point was estimated to be the most recent common ancestor (MRCA) of Sarcopterygii based on the fossils from *Latimeria* with a hard minimum age of 408 Mya and a 95% soft maximum age of 427.9 Mya [58]. The C2 calibration point was estimated as the MRCA of Teleostei from *Danio* with hard minimum age of 151.2 Mya and a 95% soft maximum age of 252.7 Mya [59]. The C3 calibration point was estimated to be the MRCA of Otophysa (~157.2–166.0 Mya, [57]). A total of 100,000 samples were used for the Markov chain Monte Carlo (MCMC) analysis [56], and the first 20% samples were discarded as burn-in. An independent rate model (clock = 2) following a lognormal distribution was applied for the MCMC search.

## Supplementary Materials

The following are available online at https://www.mdpi.com/1422-0067/20/7/1529/s1, Figure S1: Length distributions of the raw contigs assembled by Trinity. Figure S2: Length distribution of the unigenes. Figure S3: Length distribution of the unigenes with FPKM ≥ 1. Figure S4: The multiple alignments of *Scribble* isoforms in Yenyuan stream salamander and other vertebrates. Table S1: The completeness estimation of predicted protein-coding sequences from the transcriptome of Yenyuan stream salamander by BUSCOs. Table S2: The annotated state of all predicted encoding sequence based on nr, Swiss-Prot, KEGG, and KOG. Table S3: The functional annotation of the top 20 genes based on RNA expression. Table S4: The statistics of gene family clustering among the Yenyuan stream salamander and other vertebrates. Data Availability: The transcriptome assembly of the Yenyuan stream salamander has been deposited at the NCBI Genbank under the project ID of PRJNA515112.
