# Supplementary material for: The First Transcriptome Assembly of Yenyuan Stream Salamander (Batrachuperus yenyuanensis) Provides Novel Insights into Its Molecular Evolution
Source: Int J Mol Sci. 2019 Mar 27;20(7):1529. doi: 10.3390/ijms20071529 (PMC6480382; doi:10.3390/ijms20071529)
Supplement: Supplementary file 1 [file ijms-20-01529-s001.zip › Figure S4.pdf]

```
                                                  10        20        30        40        50        60        70        80
conservation
Asian Arowana Scribble                            MLKCIPLWRCNRHVESLDKRHCSLQVVPDEIYRYNRSLEELLLDANQLRDLPKPFFRLLNLRKLTLSDNEIQRLPPEVAN   80
Cattle Scribble                                   MLKCIPLWRCNRHVESVDKRHCSLQAVPEEIYRYSRSLEELLLDANQLRELPKPFFRLLNLRKLGLSDNEIQRLPPEVAN   80
Zebrafish Scribble                                MLKCIPLWRCNRHVESVDKRHCSLTAVPDEIYRYNRSLEELLLDANQLRELPKPFFRLHNLRKLGLSDNEIQKLPPDVAN   80
Coelacanth Scribble                               ................................................................................    0
Spotted gar Scribble                              M....PLWRCNRHVESVDKRHCSLQTVPEEIYRYNRSLEELLLDANQLRELPKPFFRLLNLRKLGLSDNEIQRLPPEVAN   76
Chinese softshell turtle Scribble                 ....................................................QPFFRLLNLRKLGLSDNEIQRLPPEVAN   28
Zebra finch Scribble                              ..................................................LSQPFFRLLNLRKLGLSDNEIQRLPPEVAN   30
Medaka Scribble                                   MLKCIPLWRCNRHVESVDKRHCNLHTVPDEIFRYSRSLEELLLDANQLKELPKPFFRLLNLRKLGLSDNEIQRLPPEVAN   80
Yenyuan stream salamander Scribble isoform1       ................................................................................    0
Yenyuan stream salamander Scribble isoform2       ................................................................................    0
Yenyuan stream salamander Scribble isoform3       ................................................................................    0
Yenyuan stream salamander Scribble isoform4       ................................................................................    0
Yenyuan stream salamander Scribble isoform5       ................................................................................    0
Yenyuan stream salamander Scribble isoform6       ................................................................................    0
Yenyuan stream salamander Scribble isoform7       ................................................................................    0
Yenyuan stream salamander Scribble isoform8       ................................................................................    0
Yenyuan stream salamander Scribble isoform9       ................................................................................    0
```

```
                                                  90        100       110       120       130       140       150       160
conservation
Asian Arowana Scribble                            FMQLVELDISRNDIPEIPESIKFCKALEIADFSGNPLSRLPEGFTQLRGLAHLALNDVSLQTLPNDIGNLANLVTLELRE  160
Cattle Scribble                                   FMQLVELDVSRNDIPEIPESIKFCKALEIADFSGNPLSRLPEGFTQLRSLAHLALNDVSLQALPGDVGNLANLVTLELRE  160
Zebrafish Scribble                                FTQLVELDISRNDISEIPENIKFCQSLEIADFSGNPLTRLPDGFTQLRGLAHLSLNDVSLQSLPNDIGNLSNLVTLELRE  160
Coelacanth Scribble                               ................................................................................    0
Spotted gar Scribble                              FMQLVELDISRNDIPEIPESIKFCKALEIADFSGNPLSRLPDGFTQLRSLAHLALNDVSLQALPNDIGNLANLVTLELRE  156
Chinese softshell turtle Scribble                 FMQLVELDISRNDIPEIPESIKFCKSLEIADFSGNPLSRLPEGFTQLRSLGHLALNDVSLQSLPSDIGNLANLVTLELRE  108
Zebra finch Scribble                              FMQLVELDISRNDIPEIPESIKFCKSLEIADFSGNPLSRLPEGFTQLRSLVHLALNDVSLQSLPTDIGNLANLVTLELRE  110
Medaka Scribble                                   FMQLVELDISRNDIPEIPESIKFCRALEIADFSGNPLSRLPDGFTQLRTLAHLALNDVSLQTLPNDIGNLANLVTLELRE  160
Yenyuan stream salamander Scribble isoform1       ................................................................................    0
Yenyuan stream salamander Scribble isoform2       ................................................................................    0
Yenyuan stream salamander Scribble isoform3       ................................................................................    0
Yenyuan stream salamander Scribble isoform4       ................................................................................    0
Yenyuan stream salamander Scribble isoform5       ................................................................................    0
Yenyuan stream salamander Scribble isoform6       ................................................................................    0
Yenyuan stream salamander Scribble isoform7       ................................................................................    0
Yenyuan stream salamander Scribble isoform8       ................................................................................    0
Yenyuan stream salamander Scribble isoform9       ................................................................................    0
```

```
                                                  170       180       190       200       210       220       230       240
conservation
Asian Arowana Scribble                            NLLKTLPTSLSFLVKLEQLDLGSNELEVLPDTLGALPNLRELLLDRNHLSALPPELGNLRRLVCLDVSENRLEQLPPELS  240
Cattle Scribble                                   NLLKSLPASLSFLVKLEQLDLGGNDLEVLPDTLGALPNLRELWLDRNQLSALPPELGNLRRLVCLDVSENRLEELPAELG  240
Zebrafish Scribble                                NLLKSLPSSLSFLVKLEQLDLGSNVLEVLPDTLGALPNLRELWLDRNQLSSLPPELGNLRQLVCLDVSENRLSELPTEIS  240
Coelacanth Scribble                               ................................................................................    0
Spotted gar Scribble                              NLLKSLPTSLSFLVKLEQLDLGSNELEVLPDTLGALPNLRELWLDRNHLSSLPPELGNLRRLVCLDVSENRLEQLPSELS  236
Chinese softshell turtle Scribble                 NLLKALPTSLSFLVKLEQLDLGGNDLEVLPDTLGALPNLRELWLDRNQLSALPPELGNLRRLVCLDVSENKLEHLPNELS  188
Zebra finch Scribble                              NLLKTLPTSLSFLVKLEQLDLGGNDLEVLPDTLGALPNLRELWLDRNQLSALPPELGNLRRLVCLDVSENKLEQLPNEVS  190
Medaka Scribble                                   NLLKSLPTSLSFLVKLEQLDLGSNELEDLPDTLGALPNLRELWLDRNQLSTLPEELGNLRRLVCLDVSENRLEELPSELN  240
Yenyuan stream salamander Scribble isoform1       ................................................................................    0
Yenyuan stream salamander Scribble isoform2       ................................................................................    0
Yenyuan stream salamander Scribble isoform3       ................................................................................    0
Yenyuan stream salamander Scribble isoform4       ................................................................................    0
Yenyuan stream salamander Scribble isoform5       ................................................................................    0
Yenyuan stream salamander Scribble isoform6       ................................................................................    0
Yenyuan stream salamander Scribble isoform7       ................................................................................    0
Yenyuan stream salamander Scribble isoform8       ................................................................................    0
Yenyuan stream salamander Scribble isoform9       ................................................................................    0
```

```
conservation
Asian Arowana Scribble                        GLLALTDLLLSQNLLELLPDTLGSLKQLSILKVDQNRLTHLTDSIGECENLSELVLTENLLQTLPRSMGKLKKLNNLNVD   320
Cattle Scribble                               GLVLLTDLLLSQNLLQRLPDGIGQLKQLSILKVDQNRLCEVTEAIGDCENLSELILTENLLTALPHSLGKLTKLTNLNVD   320
Zebrafish Scribble                            GLIALTDLLLSENLLEILPDSIGSLKKLSILKVNQNRLVHLTDSIGECENLTELMLTENLLQSLPRSLGKLKKLTNLNVD   320
Coelacanth Scribble                           ................................................................................     0
Spotted gar Scribble                          GLLALTDLLLSQNLLETLPDSIGCLKQLSILKVDQNRLTSLTDSIGECENLSELMLTENLLQTLPRSLGKLKKLTNLNVD   316
Chinese softshell turtle Scribble             GLVALTDLLLSQNLLESIPDGIGQLKQLSILKVDQNRLVEVTESIGDCENLSELILTENVLTALPKSLGKLTKLTNLNVD   268
Zebra finch Scribble                          GLVALTDLLLSQNLLECIPDGIGQLKQLSILKVDQNRLTDMTESIGDCENLSELILTENMLTALPKSLGKLAKLTNLNVD   270
Medaka Scribble                               GLLALTDLLLTQNLLEFVPDSIGSLKQLSILKVDQNRMTNLTDSIGECENLTELVLTENLLQSLPQSLGKLKKLTNLNVD   320
Yenyuan stream salamander Scribble isoform1   ................................................................................     0
Yenyuan stream salamander Scribble isoform2   ................................................................................     0
Yenyuan stream salamander Scribble isoform3   ................................................................................     0
Yenyuan stream salamander Scribble isoform4   ................................................................................     0
Yenyuan stream salamander Scribble isoform5   ................................................................................     0
Yenyuan stream salamander Scribble isoform6   ................................................................................     0
Yenyuan stream salamander Scribble isoform7   ................................................................................     0
Yenyuan stream salamander Scribble isoform8   ................................................................................     0
Yenyuan stream salamander Scribble isoform9   ................................................................................     0

conservation
Asian Arowana Scribble                        RNRLYSVPEELGGCTSLNVLSLRDNRLARLPAELAGATELHVLDVAGNRLRNLPFALANLNLKAMWLAENQSQPMLKFQM   400
Cattle Scribble                               RNHLEALPPEIGGCVALSVLSLRDNRLAVLPPELAHTAELHVLDVAGNRLQSLPFALTHLNLKALWLAENQAQPMLRFQT   400
Zebrafish Scribble                            RNRLSSVPAELGGCVSLNVLSLRDNRLGKLPPELANATELHVLDVAGNRLQNLPFALANLNLKAMWLAENQSQPMLKFQT   400
Coelacanth Scribble                           ...............................................SRLQNLPFALTNLNLKALWLAENQSQPMLKFQT    33
Spotted gar Scribble                          RNRLTCLPVELGGCTGLNVLSLRDNRVAKLPAELASATELHVLDVAGNRLQNLPFALTNLNLKAMWLAENQSQPMLKFQT   396
Chinese softshell turtle Scribble             RNRLTALPSEIGGCASLNVLSLRDNRLALLPPELANTTELHVLDVAGNRLQNLPFALTNLNLKALWLAENQSQPMLKFQT   348
Zebra finch Scribble                          RNRLTALPAEIGGCANLSVLSLRDNRLALLPPELANTTELHVLDVAGNRLQNLPFALTNLNLKALWLAENQSQPMLKFQT   350
Medaka Scribble                               RNRLSSVPKELGGCSSLNVLSLRDNRLGKLPAELADATELHVLDVAGNRLQNLPFSLTNLNLKAMWLAENQSQPMLKFQT   400
Yenyuan stream salamander Scribble isoform1   ................................................................................     0
Yenyuan stream salamander Scribble isoform2   ................................................................................     0
Yenyuan stream salamander Scribble isoform3   ................................................................................     0
Yenyuan stream salamander Scribble isoform4   ................................................................................     0
Yenyuan stream salamander Scribble isoform5   ................................................................................     0
Yenyuan stream salamander Scribble isoform6   ................................................................................     0
Yenyuan stream salamander Scribble isoform7   ................................................................................     0
Yenyuan stream salamander Scribble isoform8   ................................................................................     0
Yenyuan stream salamander Scribble isoform9   ................................................................................     0

conservation
Asian Arowana Scribble                        EDDEQTGEKVLTCYLLPQQPSPSL.....EDLPPNSVDDSW.TDSNLNRVSTIQFQEEPKAEEEDEETAAERKGLQRRAT   474
Cattle Scribble                               EDDAQTGEKVLTCYLLPQQPLPSL....EDPELQSSPSESW.GDAPLSRVSVIQFLEAPAGDDDPEEAAAEKRGLQRRAT   475
Zebrafish Scribble                            EDDEQTGEKVLTCYLLPQQPSSSL.....ENLLERSVDEAWPTDTNLNRVSVIQFRDDSKHEEEDDETAADKRGLQRRAT   475
Coelacanth Scribble                           EDDEKTGEKVLTCYLFPQQPSPSL.....ENLLQNSMDENW.TDTNLNRVSVIQFQEEPKPDEDDDEEAAERRGLQRRAT   107
Spotted gar Scribble                          EDDEKTGEKVLTCYLLPQQPSPSL.....ENLLQNSVDDSW.TDSNLNRVSVIQFQEETKPEAEDEDTAAERRGLQRRAT   470
Chinese softshell turtle Scribble             EDDEKTGEKVLTCYLLPQQPSPSLGKRSHENLLQNSVDESW.TDTNLNRVSVIQFLDEPRADDE.EEAGAEKRGLQRRAT   426
Zebra finch Scribble                          EDDEKTGEKVLTCYLLPQQPSPSL.....ENLLQNSVDESW.TDTNLNRVSVIQFLDEPKGDDD.EESGAERRGLQRRAT   423
Medaka Scribble                               EDDERTGEKVLTCYLLPQQPSPSL.....ENLLQSSVDDSW.TDSNLNRVSVIQFQEETKGEDEDDEAAAERRGLQRRAT   474
Yenyuan stream salamander Scribble isoform1   ................................................................................     0
Yenyuan stream salamander Scribble isoform2   ................................................................................     0
Yenyuan stream salamander Scribble isoform3   ................................................................................     0
Yenyuan stream salamander Scribble isoform4   ................................................................................     0
Yenyuan stream salamander Scribble isoform5   ................................................................................     0
Yenyuan stream salamander Scribble isoform6   ................................................................................     0
Yenyuan stream salamander Scribble isoform7   ................................................................................     0
Yenyuan stream salamander Scribble isoform8   ................................................................................     0
Yenyuan stream salamander Scribble isoform9   ................................................................................     0
```

```
                                                  480            490            500            510            520            530            540
conservation
Asian Arowana Scribble                         PHPSELKVMKKVIEERRNEPYTARPEEAELSPSTEEKRLSGLSNQ.SHDSQASNSTASAGSREDVPGQTT...AWVG...   547
Cattle Scribble                                PHPSELKVMKRGVE.RRSEATSCRPDPAPPSPSEEEKRLSTESGL.SADSHLSAGTVSQGEPEGPLAEEE..........   543
Zebrafish Scribble                             PHPSELKVMKNVIEARRNEAYTARPDEDLESPDAEEKRLSGLSNQ.SHDSQASSSTTSATSHEDKQGI.....NSTG...   546
Coelacanth Scribble                            PHPSELKVMKKVIEDRRNEAYTAKPDGDLTTPDAEEKRLSTLSNQ.SQESHASNATSSAGSEEQRPKETERGNATVVRFA   186
Spotted gar Scribble                           PHPSELKVMKKVIEERRNEGFTARPDGDDETPEREEKRLSGLSSQRSQDSQASSSTASASSQEERQGQAD....AVGVPG   546
Chinese softshell turtle Scribble              PHPSELKVMKKVIEVRRNEMYSAKPDVNPDSPDSEEKRLSAMSSR.SEDSHVSNSTVSANFREEEEVGAG..........   495
Zebra finch Scribble                           PHPSELKVMKKVIEVRRSEVNAAKADANPSSPNSEEKRLSATSNR.SEDSHLSTSTASLQREGKPRAEEE..........   492
Medaka Scribble                                PHPSELKVMKKVIEERRNEAYTSRADGADENEDQQEKRLSDLSNQ.SHDSQVSNSTLSAASHEDRLNAAP...NTQR...   547
Yenyuan stream salamander Scribble isoform1    ................................................................................     0
Yenyuan stream salamander Scribble isoform2    ................................................................................     0
Yenyuan stream salamander Scribble isoform3    ................................................................................     0
Yenyuan stream salamander Scribble isoform4    ................................................................................     0
Yenyuan stream salamander Scribble isoform5    ................................................................................     0
Yenyuan stream salamander Scribble isoform6    ................................................................................     0
Yenyuan stream salamander Scribble isoform7    ................................................................................     0
Yenyuan stream salamander Scribble isoform8    ................................................................................     0
Yenyuan stream salamander Scribble isoform9    ................................................................................     0

                                                   550            560            570            580            590            600            610            620
conservation
Asian Arowana Scribble                         ..TVDGHSQEQEGEAL.DEMEVEYIEPTVHFAEEPIIRGGDDEEEEGYGEEHEEEAEQSD.EGERP.PFPMERQRLIRKD   622
Cattle Scribble                                GLSQQEPMPATQE....EVMEETYEEPTVRFAEDTLLLPREDGE.............SEEGQPEAPWPLPGGRQRLIRKD   606
Zebrafish Scribble                             VELNDGQV.QEEEDL..DEMEVEYTEPTVHFAEEPIIRGGDEDDDY...DNDDDDAERSDEVSQTP.SFPAEKQRLIRKD   619
Coelacanth Scribble                            GEGPDRQEAEQEEVGPEDEMEVEYIEPSVHFAEETVLRDEDGEE..................EERP.VNIVEKQRLIRKD   247
Spotted gar Scribble                           EESPKGQAEEQEEEGLGDEMEVEYIEPTVHFAEEPIIRGGEEEE....EDEEEDGERSEGEEERP.AFPMEKQRLIRKD   620
Chinese softshell turtle Scribble              .........GTRPNGQVPEKQELESEPSVHFAEDTLVRSEEEDD...................DDERPFPVEKQRLIRKD   547
Zebra finch Scribble                           ........HKEAADQEEEDVEVEYNEPTVHFAEDTLIRSEDEDE...................DEERPFPVEKQRLIRKD   545
Medaka Scribble                                EDLVDGHSPQDEEEL..DEMEVEYIEPTVHFAEEPIIRGGDEDE....EEGGENGDRTDEEDEKP.SFPMEKQRLIRKD   619
Yenyuan stream salamander Scribble isoform1    ................................................................................     0
Yenyuan stream salamander Scribble isoform2    ................................................................................     0
Yenyuan stream salamander Scribble isoform3    ................................................................................     0
Yenyuan stream salamander Scribble isoform4    ................................................................................     0
Yenyuan stream salamander Scribble isoform5    ................................................................................     0
Yenyuan stream salamander Scribble isoform6    ................................................................................     0
Yenyuan stream salamander Scribble isoform7    ................................................................................     0
Yenyuan stream salamander Scribble isoform8    ................................................................................     0
Yenyuan stream salamander Scribble isoform9    ................................................................................     0

                                                       630            640            650            660            670                      680        690
conservation
Asian Arowana Scribble                         TPHYKKHFKITKLPKPEAVAALLQGFSPDST.PTRQQPPEEEEEEEEAEQQQEEEDLA.........LHPRIEELD..NSR   690
Cattle Scribble                                TPHYKKHFKISKLPQPEAVVALLQGTPPDNEGPVGTGGWHNGPHMSWAPRAEEEEEE.........EEDEAEEEEEGEAA   677
Zebrafish Scribble                             TPHYKKHFKITKLPKPETVAALLQGFSHDGLSPTAHTPENERDGEDDEEEEEDED................EEDDLHTP.   682
Coelacanth Scribble                            TPHYKKHFKITKLPKPEVVVALLQGYSSEGVPEEEEELGALRNHTEEEPADETQDGH.........TQFKYRVQSLFLTD   318
Spotted gar Scribble                           TPHYKKHFKITKLPKPEAVAALLQGLSAEVLPPAEDGEGPEVVTQPLPG.......................DELDWGESR   678
Chinese softshell turtle Scribble              TPHYKKHFKITKLPKPEAVVALLQGLSSDGVPEEEERGGCSNSVEPPEQEEAWDEDS.......................   604
Zebra finch Scribble                           TPHYKKHFKITKLPKPEAVVALLQGLNSDGEEELGGCNNNTEPRDQDEAWDEDDRKNGALSAQPSVKEEGQRRADLPSAP   625
Medaka Scribble                                TPHYKKHFKITKLPKPETVAALLQGFSPDGLNSPT......RAGE..EEDEEDDD................QSTPLHHPR   675
Yenyuan stream salamander Scribble isoform1    ................................................................................     0
Yenyuan stream salamander Scribble isoform2    ................................................................................     0
Yenyuan stream salamander Scribble isoform3    ................................................................................     0
Yenyuan stream salamander Scribble isoform4    ................................................................................     0
Yenyuan stream salamander Scribble isoform5    ................................................................................     0
Yenyuan stream salamander Scribble isoform6    ................................................................................     0
Yenyuan stream salamander Scribble isoform7    ................................................................................     0
Yenyuan stream salamander Scribble isoform8    ................................................................................     0
Yenyuan stream salamander Scribble isoform9    ................................................................................     0
```

```
                                                      700       710       720       730       740       750       760
conservation
Asian Arowana Scribble                       LTDRVNSSLQ....PVKGVSFDQVNNLLIEPARIEEEELTLTIVRQTGGLGISIAGGRGSTPYKGDDEGIFISRVSEEGP  766
Cattle Scribble                              AAAEEEKA.VASAPSVKGVSFDQANNLLIEPARIEEEELTLTIVRQTGGLGISIAGGKGSTPYKGDDEGIFISRVSEEGP  756
Zebrafish Scribble                           ......FQHRLDAAELEDIRMSQMNSSL.QPVKL.....TLSILRQTGGLGISIAGGKGSTPYKGDDEGIFISRVSEEGP  750
Coelacanth Scribble                          LMMMGPPR..SSWNLKKGVSFDQVNNLLIEPARIEEEELTLTIHRQTGGLGISIAGGKGSTPYKGDDEGIFISRVSEEGP  396
Spotted gar Scribble                         LSDRGSASLQ....PVKGVSFDQVNNLLIEPARIEEEELTLTILRQTGGLGISIAGGKGSTPYKGDDEGIFISRVSEEGP  754
Chinese softshell turtle Scribble            ....VKDGALPTQPSVKGVSFDQANNLLIEPARIEEEELTLTILRQTGGLGISIAGGKGSTPYKGDDEGIFISRVSEEGP  680
Zebra finch Scribble                         VSRQPGSGVQLWSPAVTGVSFDQANNLLIEPARIEEEELTLTIVRQTGGLGISIAGGKGSTPYKGDDEGIFISRVSEEGP  705
Medaka Scribble                              MEELGDSRLQVNSSQVKGVSFDQVNNLLIEPARIEEEEHTITIQRQTGGLGISIAGGKGSTPYKGDDEGIFISRVSAEGP  755
Yenyuan stream salamander Scribble isoform1  ................................................................................    0
Yenyuan stream salamander Scribble isoform2  ....................................................................GIFISRVAEEGP   12
Yenyuan stream salamander Scribble isoform3  ................................................................................    0
Yenyuan stream salamander Scribble isoform4  ................................................................................    0
Yenyuan stream salamander Scribble isoform5  ....................................................................GIFISRVAEEGP   12
Yenyuan stream salamander Scribble isoform6  ................................................................................    0
Yenyuan stream salamander Scribble isoform7  ................................................................................    0
Yenyuan stream salamander Scribble isoform8  ................................................................................    0
Yenyuan stream salamander Scribble isoform9  ....................................................................GIFISRVAEEGP   12

                                                  770       780       790       800       810       820       830       840
conservation
Asian Arowana Scribble                       AARAGVKVGDKLLEVNGVDLHEAEHQQAVEALRSSGASVTMTVLRERMVEPENAVTTTPLRPEHDYLPRERRSG...SSP  843
Cattle Scribble                              AARAGVRVGDKLLEVNGVALQDAEHQQAVEALRGAGATVHMRLWRERMVEPENAVTVTALRPEDDYSPRERRGG...GLR  833
Zebrafish Scribble                           AARAGVKVGDKLLEVNGVDLHGAEHHTAVEALRNSGAAVVMTVLRERMVEPENAITTTPLRPEDDYFPRERRSS...GLP  827
Coelacanth Scribble                          AARAGVRIGDKLLEVNGVDLHNAEHHKAVEALRSSGSAVTMVVLRERMVEPENAITVTPLRPEDDYYPRERRSG....LR  472
Spotted gar Scribble                         AARAGVKVGDKLLEVNGVDLHDAEHHKAVEALRSSGSAVSMTVLRERMVEPENAITTTPLRPEDDYFPRERRSGLPFT..  832
Chinese softshell turtle Scribble            AARAGVRVGDKLLEVNGVALHSAEHHVAVDALRGSGSSVSMTVLRERMVEPENAITVTPLRPEDDYSPREPRGGLRFPER  760
Zebra finch Scribble                         AARAGVRVGDKLLERSGFVPACNEHQVAEKTKKESGSSVSMTVLRERMVEPENTITVTPLRPEEDYSPRERRGGLRFPEG  785
Medaka Scribble                              AARAGVKVGDKLLEVNGVDLHEAEHHTAVEALRSSGATVSMTVLREHMVEPENAITTTPLRPEDDYFPRERRSS...GIA  832
Yenyuan stream salamander Scribble isoform1  ................................................................................    0
Yenyuan stream salamander Scribble isoform2  AARAGVRVGDKLLEVNGVDLHNAEHHKAVEALRSSGTSVSMSVLRERMVEPENAITVTPLRPEDDYYPRERRSGMRFPED   92
Yenyuan stream salamander Scribble isoform3  ............................................ATRLLEEEAESYLTQPLK..................   18
Yenyuan stream salamander Scribble isoform4  ............................................ATRLLEEEAESYLTQPLK..................   18
Yenyuan stream salamander Scribble isoform5  AARAGVRVGDKLLEVNGVDLHNAEHHKAVEALRSSGTSVSMSVLRERMVEPENAITVTPLRPEDDYYPRERRSGMRFPED   92
Yenyuan stream salamander Scribble isoform6  ............................................ATRLLEEEAESYLTQPLK..................   18
Yenyuan stream salamander Scribble isoform7  ............................................ATRLLEEEAESYLTQPLK..................   18
Yenyuan stream salamander Scribble isoform8  ............................................ATRLLEEEAESYLTQ.....................   15
Yenyuan stream salamander Scribble isoform9  AARAGVRVGDKLLEVNGVDLHNAEHHKAVEALRSSGTSVSMSVLRERMVEPENAITVTPLRPEDDYYPRERRSGMRFPED   92

                                                      850       860       870       880       890       900       910       920
conservation
Asian Arowana Scribble                       FCTEP...CFSQGTRQRLSTCLVRNDKGLGFSIAGGKGSTPYRVGDMGIFISRIAEGGAADRDSILQVGDRVISINGVDM  920
Cattle Scribble                              LPLLPPEPPG..PPRQRHVACLVRSEKGLGFSIAGGKGSTPYRAGDGGIFISRIAEGGAAHRAGTLQVGDRVLSINGVDM  911
Zebrafish Scribble                           FLLDPDCPAVSTGPAQRLATCLIRNDKGLGFSIAGGKGSTLYRVGDTGIFISRIAEGGAAHRDNILQVGDRVISINGVDM  907
Coelacanth Scribble                          FASEE...PEIPVVREQFSTCLMRNEKGLGFSIAGGKGSTPYKAGDTGIFISRIAEGGAAHRDGILQVGDRVISINGVSM  549
Spotted gar Scribble                         .....PNSPGASGPCQRLSTCLVRNDKGLGFSIAGGKGSTPYHTGDTGIFISRIAEGGAAHRDGTLHVGDRVITINGVDM  907
Chinese softshell turtle Scribble            ........PEGAPPTERFSTCLVRNEKGLGFSIAGGKGSTPYKAGDMGIFISRIAEGGAAHRDGTLRVGDRVISINGVDM  832
Zebra finch Scribble                         ........PEAAPATERFSTCLMRNEKGLGFSIAGGKGSTPYRAGDMGIFISRIAEGGAAHRDGILRVGDRVISINGVDM  857
Medaka Scribble                              FNLEP....TTSGPQQRLTTCLIRNDKGLGFSIAGGKGSTPYRTGDTGIYISRIAEGGAAHRDSVLCVGDRVISINGVDM  908
Yenyuan stream salamander Scribble isoform1  ................................................................................    0
Yenyuan stream salamander Scribble isoform2  VEEEEEETAVVPVPTERFSTCLMRNEKGLGFSIAGGKGSTPYRVGDTGIFISRIAEAGAAHKDGKLNVGDRVISINGVDM  172
Yenyuan stream salamander Scribble isoform3  ............................LDYKTLAALPSTSLQK......VNRLADSDT.....................   43
Yenyuan stream salamander Scribble isoform4  ............................LDYKTLAALPSTSLQK......VNRLADSDT.....................   43
Yenyuan stream salamander Scribble isoform5  VEEEEEETAVVPVPTERFSTCLMRNEKGLGFSIAGGKGSTPYRVGDTGIFISRIAEAGAAHKDGKLNVGDRVISINGVDM  172
Yenyuan stream salamander Scribble isoform6  ............................LDYKTLAALPSTSLQK......VNRLADSDT.....................   43
Yenyuan stream salamander Scribble isoform7  ............................LDYKTLAALPSTSLQK......VNRLADSDT.....................   43
Yenyuan stream salamander Scribble isoform8  ................................................................................   15
Yenyuan stream salamander Scribble isoform9  VEEEEEETAVVPVPTERFSTCLMRNEKGLGFSIAGGKGSTPYRVGDTGIFISRIAEAGAAHKDGKLNVGDRVISINGVDM  172
```

```
                                                      930           940           950                                 960           970
conservation
Asian Arowana Scribble                            TDARHDQAVALLTGTSPTITLLVERDQGDT.............PRARPHSPPPPQPSE.SLDQEE.......SQANHL.N    978
Cattle Scribble                                   TEARHDHSVSLLTAASPTIALLLCSSCGRC.......AGQRLSRDAHPHSPPPPTATP.ATPGEPGPLR....LPPSLLA    979
Zebrafish Scribble                                TEARHDQAVALLTGTSPTITLVVDREQSSV.......GGAS..PRTRPHSPPPPEPSD.SPEQEDGGDE...HLGNHL.N    973
Coelacanth Scribble                               TDARHDQAVALLTGTSPTIPLLVERETLEPPSD.GEAGASPALYRARPHSPPPPPGQADPSEGENRNEEEIVAQSNHISS    628
Spotted gar Scribble                              TEARHDQAVTLLTGTSPTITLLVERDVGDPAADSAGLGGSPALARARPHSPPPPEPCD.TPDQEDISEDRAYPQDNHL.S    985
Chinese softshell turtle Scribble                 TEARHDQAVALLTASSPTIALLVEREGAEQ......LSEGDSPVAPRAHSPPPPSSHGESPPEETALL.....QRDHL.S    900
Zebra finch Scribble                              TEARHDQAVALLTAASPTIVLLVEREGAEQ........PGEGGSPRHALPPAIPRTWDDIIISTPASTVISAAVNQYL.S    928
Medaka Scribble                                   TEARHDQAVALLTGTSPTISLVVERDPKAS.......MGSPGQSRARAHSPPPPEPSD.SPDQDEEGLQ.....GNHL.G    974
Yenyuan stream salamander Scribble isoform1       ................................................................................      0
Yenyuan stream salamander Scribble isoform2       TEARHDQAVALLTGSSPTITLLVEREIIAPGTE.ADLGASPALQRTRPHSPPPPSHLD.AGEEDDGQEADISQQRNHL.A    249
Yenyuan stream salamander Scribble isoform3       ......HTLAMETDCQVLQGLLPSTDSGGL...........................................QSQGLAS     74
Yenyuan stream salamander Scribble isoform4       ......HTLAMETDCQVLQGLLPSTDSGGL...........................................QSQGLAS     74
Yenyuan stream salamander Scribble isoform5       TEARHDQAVALLTGSSPTITLLVEREIIAPGTE.ADLGASPALQRTRPHSPPPPSHLD.AGEEDDGQEADISQQRNHL.A    249
Yenyuan stream salamander Scribble isoform6       ......HTLAMETDCQVLQGLLPSTDSGGL...........................................QSQGLAS     74
Yenyuan stream salamander Scribble isoform7       ......HTLAMETDCQVLQGLLPSTDSGGL...........................................QSQGLAS     74
Yenyuan stream salamander Scribble isoform8       ................................................................................     15
Yenyuan stream salamander Scribble isoform9       TEARHDQAVALLTGSSPTITLLVEREIIAPGTE.ADLGASPALQRTRPHSPPPPSHLD.AGEEDDGQEADISQQRNHL.A    249

                                                  980       990       1000      1010      1020      1030      1040      1050
conservation
Asian Arowana Scribble                            RALEDEYPVEEVTLVKTGGPLGLSIVGGSDHASHPFGVTEPGVFISKVVLQGLACNSGLRVGDRILEVNGIDLRHATHQE   1058
Cattle Scribble                                   AALEGPYPVEEICLPRAGGPLGLSIVGGSDHSSHPFGVQEPGVFISKVLPRGLAARSGLRVGDRILAVNGQDVREATHQE   1059
Zebrafish Scribble                                CPMEDEYPIEEVTLIKAGGPLGLSIVGGSDHASHPFGINEPGVFISKVIPNGLASQSGLRVGDRILEVNSIDLRHATHQE   1053
Coelacanth Scribble                               REMEDDYPVEVRHL...AGILSIELAGKSDDNFSSVSLGPPCVYIRKVIPQGLAARSGLRVGDRILEVNAIDLRHATHQE    705
Spotted gar Scribble                              RQLEDEYPIEEVTLMKAGGPLGLSIVGGSDHASHPFGISEPGVFISKVIPQGLAAQSGLRVGDRILEVNSIDLRHATHQE   1065
Chinese softshell turtle Scribble                 KGLEDQYPIEEICLIKAGGPLGLSIVGGSDHSSHPFGIQEPGVFISKVIPHGLASRSGLRVGDRILEVNTIDLRHATHQE    980
Zebra finch Scribble                              KGLEDQYPVEEIRLVKAGGPLGLSIVGGSDHSSHPFGIHEPGVFISKVIPRGLASRSGLRVGDRILEVNGIDLRHATHQE   1008
Medaka Scribble                                   .QMEDQYPIEEVTLLKSGGPLGLSIVGGSDHASHPFGVNEPGVFISKVIPHGLASQSGLRVGDRILEVNSIDLRHATHQE   1053
Yenyuan stream salamander Scribble isoform1       ................................................................................      0
Yenyuan stream salamander Scribble isoform2       RGMEDEYPMEEVLLLKAGGPLGLSIVGGSDHSSHPFGVHEPGVFISKVIPQGLAARSGLRVGDRILEVNTIDLRHATHQE    329
Yenyuan stream salamander Scribble isoform3       AAAPQELVVNCVNICPAA..............................VPEGAPLYHTVQLEEKTAAAG....EQTAHST    120
Yenyuan stream salamander Scribble isoform4       AAAPQELVVNCVNICPAA..............................VPEGAPLYHTVQLEEKTAAAG....EQTAHST    120
Yenyuan stream salamander Scribble isoform5       RGMEDEYPMEEVLLLKAGGPLGLSIVGGSDHSSHPFGVHEPGVFISKVIPQGLAARSGLRVGDRILEVNTIDLRHATHQE    329
Yenyuan stream salamander Scribble isoform6       AAAPQELVVNCVNICPAA..............................VPEGAPLYHTVQLEEKTAAAG....EQTAHST    120
Yenyuan stream salamander Scribble isoform7       AAAPQELVVNCVNICPAA..............................VPEGAPLYHTVQLEEKTAAAG....EQTAHST    120
Yenyuan stream salamander Scribble isoform8       ................................................................................     15
Yenyuan stream salamander Scribble isoform9       RGMEDEYPMEEVLLLKAGGPLGLSIVGGSDHSSHPFGVHEPGVFISKVIPQGLAARSGLRVGDRILEVNTIDLRHATHQE    329

                                                  1060      1070      1080      1090      1100      1110      1120      1130
conservation
Asian Arowana Scribble                            AVRALLSSKHEIRMLVRRDPAPPGMQEIEIQKYPGEKLGISIRGGAKGHAGNPFDPTDEGVFISKVSSGGAAARDGRLQV   1138
Cattle Scribble                                   AVSALLRPCLELVLLVRRDPPPPGMRELCIQKAPGERLGISIRGGAKGHAGNPCDPTDEGIFISKVSPTGAAGRDGRLRV   1139
Zebrafish Scribble                                AVRALLSNKQEIRMLVRRDPSPPGMQEIVIHKQPGEKLGISIRGGAKGHAGNPFDPTDEGIFISKVSSNGAAARDGRLRV   1133
Coelacanth Scribble                               AVKALLSNKQEIRMLVRRDPPPPGMAVQEKKSEPSVCIGQTQNPDISHNPGNIFKLRDLLFLPSQVSSSGAAARDGRLKV    785
Spotted gar Scribble                              AVRALLSNKQEIRMLVRRDPAPPGMQDIVIEKLAGEKLGISIRGGAKGHAGNPFDPTDEGIFISKVSSSGAAARDGRLQV   1145
Chinese softshell turtle Scribble                 AVNALLSSTQALSMLVRRDPPPPGMQEICIEKAPGEKLGISIRGGAKGHAGNPFDPTDEGIFISKVSSSGAAARDGRLKV   1060
Zebra finch Scribble                              AVNALLSNTQELSMLVRRDPPPPGMQEICIEKAPGEKLGISIRGGAKGHAGNPFDPTDEGIFISKVSSSGAAARDGRLQV   1088
Medaka Scribble                                   AVRSLLANKQEIRMLVRRDPSPPGMQEVVIQKQPGEKLGISIRGGAKGHAGNPFDATDEGVFISKVSSTGAAARDGRLQV   1133
Yenyuan stream salamander Scribble isoform1       .....................................................YSPTTQQQ...................      8
Yenyuan stream salamander Scribble isoform2       AVNALLSNSQEIRMLVRRDPPPPGMQEISIDKCPGEKLGISIRGGAKGHAGNPFDPTDEGIFISRVSSSGAAARDGRLRV    409
Yenyuan stream salamander Scribble isoform3       SVRPTSESGTETAHHLPQQDLKPRTPE.........................PFAPTPETL................QQE    159
Yenyuan stream salamander Scribble isoform4       SVRPTSESGTETAHHLPQQDLKPRTPE.........................PFAPTPETL................QQE    159
Yenyuan stream salamander Scribble isoform5       AVNALLSNSQEIRMLVRRDPPPPGMQEISIDKCPGEKLGISIRGGAKGHAGNPFDPTDEGIFISRVSSSGAAARDGRLRV    409
Yenyuan stream salamander Scribble isoform6       SVRPTSESGTETAHHLPQQDLKPRTPE.........................PFAPTPETL................QQE    159
Yenyuan stream salamander Scribble isoform7       SVRPTSESGTETAHHLPQQDLKPRTPE.........................PFAPTPETL................QQE    159
Yenyuan stream salamander Scribble isoform8       ................................................................................     15
Yenyuan stream salamander Scribble isoform9       AVNALLSNSQEIRMLVRRDPPPPGMQEISIDKCPGEKLGISIRGGAKGHAGNPFDPTDEGIFISRVSSSGAAARDGRLRV    409
```

```
                                              1140      1150      1160      1170      1180      1190      1200      1210
conservation
Asian Arowana Scribble                        GMRILEVNNQSLLGMSHTEAVRVLRAAGDSLIILVCDGFDPRDAAAVESSPGIIANPF...IVRKNSMESISSIDRDMSP  1215
Cattle Scribble                               GLRLLEVNQQSLLGLTHGEAVQLLRSVGDTLTVLVCDGFDTSAATP.EVSPGIIANPFAAGLGRRNSLESISSIDRELSP  1218
Zebrafish Scribble                            GMRILEVGNNSLLGMTHTEAVRVLRASGDSLVMLICDGFDPKSASTIEASPGVIANPFAAGIVRKNSMESISSVDRDLSP  1213
Coelacanth Scribble                           GVRILEVNNHSLLGMTHTEAVRILRSIGDTLLVLVCDGFDPKAAAAIEASPGIIANPFVAGMGRKNSMESISSIDRDLSP   865
Spotted gar Scribble                          GMRILEVNGHSLLGMTHTEAVRTLRGVGDTLAVLVCDGFDPRDAAAVEASPGVIANPFAAGIGRKNSMESISSIDRELSP  1225
Chinese softshell turtle Scribble             GLRILEVNHQSLLGMTHTEAVQILRSVGDKLLVLVCDGFDPKAAAAIEMSPGIIANPFAAGIGRKNSLESISSIDRDLTP  1140
Zebra finch Scribble                          GMRILEVNHQSLLGMTHTEAVQVLRGVGDALLVLVCDGFDPKAAAAIEMSPGIIANPFAAGIGRKNSLESISSIDRDLSP  1168
Medaka Scribble                               GMRILEVNNHSLLGMTHTEAVRVLRAVGDSLSMLVCDGFDPQKVTSVEASPGIIANPFAAGIVRKNSMESISSIDRDLSP  1213
Yenyuan stream salamander Scribble isoform1   ................................................................................     8
Yenyuan stream salamander Scribble isoform2   GMRILEVNHQSLLGMTHTEAVQILRAVGDTLLVLVCDGFDPRAAAAIEVSPGIIANPFASGIARKNSMESISSIDRDMSP   489
Yenyuan stream salamander Scribble isoform3   GEEETLVDSQPIL.............................FSENPFVVAN.......RKGKAGSGACLGG....   195
Yenyuan stream salamander Scribble isoform4   GEEETLVDSQPIL.............................FSENPFVVAN.......RKGKAGSGACLGG....   195
Yenyuan stream salamander Scribble isoform5   GMRILEVNHQSLLGMTHTEAVQILRAVGDTLLVLVCDGFDPRAAAAIEVSPGIIANPFASGIARKNSMESISSIDRDMSP   489
Yenyuan stream salamander Scribble isoform6   GEEETLVDSQPIL.............................FSENPFVVAN.......RKGKAGSGACLGG....   195
Yenyuan stream salamander Scribble isoform7   GEEETLVDSQPIL.............................FSENPFVVAN.......RKGKAGSGACLGG....   195
Yenyuan stream salamander Scribble isoform8   ................................................................................    15
Yenyuan stream salamander Scribble isoform9   GMRILEVNHQSLLGMTHTEAVQILRAVGDTLLVLVCDGFDPRAAAAIEVSPGIIANPFASGIARKNSMESISSIDRDMSP   489
```

```
                                                  1220      1230      1240      1250      1260      1270      1280      1290
conservation
Asian Arowana Scribble                        EEMAIMQK....EVEMVQDTPRWDKEDIEDVERMRLEREEANRLLEKEAETFGTGPLKLDYRTLAALPTTSLQ.KVMRGL  1290
Cattle Scribble                               EGPGK.......EKELPGQIPLWGPEAMSAPQ.................GRSPESLKPACRALAATPGAGSMQRVLPAA  1273
Zebrafish Scribble                            EEIDIMQK....EVEMVRETSQWEREEMEKVERMRLEREEATRLLEEETESLSSGPLKLDYKTLAALPTTSLQ.KVNRAS  1288
Coelacanth Scribble                           EEMEIIQK....EMEMVRETTRWEREEMEKV.................SYNTEPLKLDYKTLAALPATSLQ.KVNRGS   921
Spotted gar Scribble                          EEMDIIQK....EVEMVREASQWEREEIEKV.................NFSPGPLKLDYKTLAALPTTSLQ.KVTRSS  1281
Chinese softshell turtle Scribble             EELEILQK....EMEMVREATQWEREEMEKV.................NISSEPLRLDYRTLAALPTNNFP.RVTRAV  1196
Zebra finch Scribble                          EELEILQKVAGSTLQMFPQLNLAQKQGDE..........................PLRLDYRTLAALPSSGLQ.RGNRAA  1221
Medaka Scribble                               EEIDIMQK....ESEMVRETSQWEREEMEKV.................NISTGPLKLDYKTLAALPTSSLQ.KINRAP  1269
Yenyuan stream salamander Scribble isoform1   ...........................................................P........AAPRSVQLSGRETRFA    25
Yenyuan stream salamander Scribble isoform2   EEIEILQK....EMEMVRETSQWEKEELEKV.................SYLTQPLKLDYKTLAALPSTSLQ.KVNRTP   545
Yenyuan stream salamander Scribble isoform3   ...........................................................P.PVGYGKSGVLKTNMFS...KKTP   216
Yenyuan stream salamander Scribble isoform4   ...........................................................P.PVGYGKSGVLKTNMFS...KKTP   216
Yenyuan stream salamander Scribble isoform5   EEIEILQK....EMEMVRETSQWEKEELEKV.................SYLTQPLKLDYKTLAALPSTSLQ.KVNRTP   545
Yenyuan stream salamander Scribble isoform6   ...........................................................P.PVGYGKSGVLKTNMFS...KKTP   216
Yenyuan stream salamander Scribble isoform7   ...........................................................P.PVGYGKSGVLKTNMFS...KKTP   216
Yenyuan stream salamander Scribble isoform8   ...........................................................PLKLDYKTLAALPSTSLQ.KVNRTP    39
Yenyuan stream salamander Scribble isoform9   EEIEILQK....EMEMVRETSQWEKEELEKV.................SYLTQPLKLDYKTLAALPSTSLQ.KVNRTP   545
```

```
                                                           1300      1310      1320      1330      1340      1350      1360
conservation
Asian Arowana Scribble                        SSE.PQGVHSPSRDIAGSPSS.PASLHFVSTASPKEN..IPTRPGVIQPLSRMWSNS.TVNQDTRNSPKAFHHGGVPATS  1365
Cattle Scribble                               SGE.M.......AEPPCSPSHQQ.........................PPSPPSPAE..........LPANV  1302
Zebrafish Scribble                            PSE.PFRIDSPVRDAAHSPHNSQSNIHFPSNANTKDN..ASTKPGAIQPLSRVRPPVSPASQDGHSSPNPFQHG.LSPIN  1364
Coelacanth Scribble                           SSESPALNSPVKRDSPHSPTPQLASINFLSTHNDS....KSTKPGVIQPLSRVRPNMAPASQEGRDSPNPFQQP.EPESD   996
Spotted gar Scribble                          SSE.PMGLSSPSKDALYSPSSQPASINFLSNAAPKEN..KSTKPGVIQPLSRLWQNAPPASQDGQSSPSSFQRG.LPLTE  1357
Chinese softshell turtle Scribble             SDG.ASL.....RLLPLLSLSLQ.....................LRPHA.......PPSPED..........VPVNV  1229
Zebra finch Scribble                          AAG.SMQDSSMT...PRGPS............................PPSPPSPDE..........VPINV  1251
Medaka Scribble                               STD.SPRTDSPIREAPYSPIIQPANIHYTSTPTTKDDTLSSTRPGAIQPVGRVRQGTSPSTPDGH.SPNPFQHG.PSPFN  1346
Yenyuan stream salamander Scribble isoform1   SIN.FLSQSD..........SQS.................TKPGAIQPHSWVRQTVTPPSSEGRN.SPNPFQQH.ENSSN    75
Yenyuan stream salamander Scribble isoform2   SID.AVSGSHVKREAPYSPTTQQ.................TKPGAIQPHSWVRQTVTPPSSEGRNSPNPFQQH.ENSSN   605
Yenyuan stream salamander Scribble isoform3   SID.AVSGSHVKREAPYSPTTQQ.................TKPGAIQPHSWVRQTVTPPSSEGRNSPNPFQQH.ENSSN   276
Yenyuan stream salamander Scribble isoform4   SID.AVSGSHVKREAPYSPTTQQ.................TKPGAIQPHSWVRQTVTPPSSEGRNSPNPFQQH.ENSSN   276
Yenyuan stream salamander Scribble isoform5   SID.AVSGSHVKREAPYSPTTQQ.................TKPGAIQPHSWVRQTVTPPSSEGRNSPNPFQQH.ENSSN   605
Yenyuan stream salamander Scribble isoform6   SID.AVSGSHVKREAPYSPTTQQASINFLSQSDS.....QSTKPGAIQPHSWVRQTVTPPSSEGRNSPNPFQQH.ENSSN   289
Yenyuan stream salamander Scribble isoform7   SID.AVSGSHVKREAPYSPTTQQASINFLSQSDS.....QSTKPGAIQPHSWVRQTVTPPSSEGRNSPNPFQQH.ENSSN   289
Yenyuan stream salamander Scribble isoform8   SID.AVSGSHVKREAPYSPTTQQ..........................................................    61
Yenyuan stream salamander Scribble isoform9   SID.AVSGSHVKREAPYSPTTQQ.................TKPGAIQPHSWVRQTVTPPSSEGRNSPNPFQQH.ENSSN   605
```

```
                                                        1370      1380      1390      1400      1410      1420      1430      1440
conservation
Asian Arowana Scribble                            PAYQPQAPSPRSPDDFPINIKQAYKAFAAVPPSLALLDPPQEPVSQ..RNNIQPR..SPVVEPELDNDVFESEMKANSGV 1441
Cattle Scribble                                   KQAY.....................................RTF.....AAVPGL..HPPLDTPA............... 1323
Zebrafish Scribble                                SQTT.....................................DLYSP..RNNVSAKQPSPEFQPELNNEVFMDECDGEGS. 1404
Coelacanth Scribble                               THST.....................................EPFIQ..KNTVRPK.......PEA............... 1015
Spotted gar Scribble                              SHTS.....................................EPFVQTRKTNALPL..QP..SPEV............... 1381
Chinese softshell turtle Scribble                 KQAY.....................................KTF.....AAVPQS..HSLLEPQM............... 1250
Zebra finch Scribble                              KQAY.....................................KTF.....AAVPLS..QPLLGTQV............... 1272
Medaka Scribble                                   SQTS.....................................DLYGV..RNNFHHRQSSP....ES............... 1368
Yenyuan stream salamander Scribble isoform1       MHNSPGPSTPLSPDEFPVNVKQAYKTFAAVPVAQIVLE.QQDVFIP..RTPERPN..AP...SEV...............  132
Yenyuan stream salamander Scribble isoform2       MHNS.....................................DVFIP..RTPERPN..AP...SEV...............  626
Yenyuan stream salamander Scribble isoform3       MHNS.....................................DVFIP..RTPERPN..AP...SEV...............  297
Yenyuan stream salamander Scribble isoform4       MHNSPGPSTPLSPDEFPVNVKQAYKTFAAVPVAQIVLE.QQDVFIP..RTPERPN..AP...SEV...............  333
Yenyuan stream salamander Scribble isoform5       MHNS.....................................DVFIP..RTPERPN..AP...SEV...............  626
Yenyuan stream salamander Scribble isoform6       MHNS.....................................DVFIP..RTPERPN..AP...SEV...............  310
Yenyuan stream salamander Scribble isoform7       MHNS.....................................DVFIP..RTPERPN..AP...SEV...............  310
Yenyuan stream salamander Scribble isoform8       .........................................DVFIP..RTPERPN..AP...SEV...............   78
Yenyuan stream salamander Scribble isoform9       MHNS.....................................DVFIP..RTPERPN..AP...SEV...............  626

                                                            1450      1460      1470      1480      1490      1500      1510      1520
conservation
Asian Arowana Scribble                            RAGAAPISSRRSFSSDRRDYLSLAAVPRLSRTSLELQSPASTGRGSPEQRSFRDRQKYFEIDVKQQTPNTKPKPRVSLVG 1521
Cattle Scribble                                   ....................................QHPMLGPTASPEQLSFRERQKYFELEVRVPQAE.GPPKRVSLVG 1366
Zebrafish Scribble                                .......GPRPSLLSDRSEYRSLAAVPRLSRPSVEPKTPSPLGRHSPEQRSFKDRQKYFEIDVKQQTPD.KPKPRISLVG 1476
Coelacanth Scribble                               ......................................SSPRGLDSPEQRSFRERQKYFEIDVKQQQAE.KPPKRVSLVG 1056
Spotted gar Scribble                              ......................................PTSSGLESPEQRSFRDRQKYFEIDMKQQTLDSKPKPRVSLVG 1423
Chinese softshell turtle Scribble                 ......................................LSPGGLHSPEQRSFRERQKYFEMEVKQQQVE.KPPKRVSLVG 1291
Zebra finch Scribble                              ....................................AVHSPGGQHSPEQRSFRERQKYFEIEVKQQHLD.KPPKRVSLVG 1315
Medaka Scribble                                   ......................................PSPSGKDGPEQRSFKDRQKYFEIDVKQQTPE.KPKPRVSLVG 1409
Yenyuan stream salamander Scribble isoform1       ......................................STPTGLDSPEQRSFRERQKYFEIDVKQQLSE.KPPKRVSLVG  173
Yenyuan stream salamander Scribble isoform2       ......................................STPTGLDSPEQRSFRERQKYFEIDVKQQLSE.KPPKRVSLVG  667
Yenyuan stream salamander Scribble isoform3       ......................................STPTGLDSPEQRSFRERQKYFEIDVKQQLSE.KPPKRVSLVG  338
Yenyuan stream salamander Scribble isoform4       ......................................STPTGLDSPEQRSFRERQKYFEIDVKQQLSE.KPPKRVSLVG  374
Yenyuan stream salamander Scribble isoform5       ......................................STPTGLDSPEQRSFRERQKYFEIDVKQQLSE.KPPKRVSLVG  667
Yenyuan stream salamander Scribble isoform6       ......................................STPTGLDSPEQRSFRERQKYFEIDVKQQLSE.KPPKRVSLVG  351
Yenyuan stream salamander Scribble isoform7       ......................................STPTGLDSPEQRSFRERQKYFEIDVKQQLSE.KPPKRVSLVG  351
Yenyuan stream salamander Scribble isoform8       ......................................STPTGLDSPEQRSFRERQKYFEIDVKQQLSE.KPPKRVSLVG  119
Yenyuan stream salamander Scribble isoform9       ......................................STPTGLDSPEQRSFRERQKYFEIDVKQQLSE.KPPKRVSLVG  667

                                                            1530      1540      1550      1560      1570      1580      1590
conservation
Asian Arowana Scribble                            EDDLKKMKEEEARRIEQCAQDYLLDE..DEEDEEDLAKQMTEIKAQGKVLLDGVEYKIESVASQTSSSPRP..CVTPPSY 1597
Cattle Scribble                                   ADDLRKMQEEEARKLQQKRAQMLREAAANTGLPEADAPEDEEPEEEPPWAGQG...............PMA......... 1422
Zebrafish Scribble                                EDDLKKMKEEEAKRIEQRAREFMLDE.DEEEEEEDLAKQVAHMKAHGKVVLDGVEYKVESLGS.....PTSRQCATPPNY 1550
Coelacanth Scribble                               EDDLKKMKEEEARKLQQKAREVLLEDEEEEDEDEDVVKRMSDISSHGSVIIEGVEYKVERVDGRGSRSPLT.....GLNY 1131
Spotted gar Scribble                              EDDLKKMKEEEVRKMEQRAQELLLDE.EDEEEEEDLAKQMAEMKAQGKVVIDGVEYKIERVDGHSSTSPTTGYCTAKPVE 1502
Chinese softshell turtle Scribble                 EDDLKKMKEEEARKLQQKRALLLEEE..EEEEEEDAGK..PDMSMQSSVIIEGVEYKIKRLNGRSIQSPAT......... 1358
Zebra finch Scribble                              EDDLKKMKEEEARKLQQQRALLLEEEAEEDEEERQVPEAMQAATA..........WQVSSRGPTASLSP........... 1374
Medaka Scribble                                   EDDLKKMREEEERKFEQRAREYLLDEDDEEDDEEDLAKQVAHMKASGKVLLDGVEYKVEPISS.....PSQHYNLTPQRY 1484
Yenyuan stream salamander Scribble isoform1       EDDLKKMKEEEFRKLQQKRQMLLQEV..DEEDEEDVVKKMADLSSQSSIYIEGVEYKIERIDGRSQPSPVP.........  242
Yenyuan stream salamander Scribble isoform2       EDDLKKMKEEEFRKLQQKRQMLLQEV..DEEDEEDVVKKMADLSSQSSIYIEGVEYKIERIDGRSQPSPVP.........  736
Yenyuan stream salamander Scribble isoform3       EDDLKKMKEEEFRKLQQKRQMLLQEV..DEEDEEDVVKKMADLSSQSSIYIEGVEYKIERIDGRSQPSPVP.........  407
Yenyuan stream salamander Scribble isoform4       EDDLKKMKEEEFRKLQQKRQMLLQEV..DEEDEEDVVKKMADLSSQSSIYIEGVEYKIERIDGRSQPSPVP.........  443
Yenyuan stream salamander Scribble isoform5       EDDLKKMKEEEFRKLQQKRQMLLQEV..DEEDEEDVVKKMADLSSQSSIYIEGVEYKIERIDGRSQPSPVP.........  736
Yenyuan stream salamander Scribble isoform6       EDDLKKMKEEEFRKLQQKRQMLLQEV..DEEDEEDVVKKMADLSSQSSIYIEGVEYKIERIDGRSQPSPVP.........  420
Yenyuan stream salamander Scribble isoform7       EDDLKKMKEEEFRKLQQKRQMLLQEV..DEEDEEDVVKKMADLSSQSSIYIEGVEYKIERIDGRSQPSPVP.........  420
Yenyuan stream salamander Scribble isoform8       EDDLKKMKEEEFRKLQQKRQMLLQEV..DEEDEEDVVKKMADLSSQSSIYIEGVEYKIERIDGRSQPSPVP.........  188
Yenyuan stream salamander Scribble isoform9       EDDLKKMKEEEFRKLQQKRQMLLQEV..DEEDEEDVVKKMADLSSQSSIYIEGVEYKIERIDGRSQPSPVP.........  736
```

```
                                              1600                1610                1620                1630                1640                1650                1660                1670
conservation
Asian Arowana Scribble                        PATPPSS....GPSSVDGRGDSQRNSLEDSFRVEQRPNSMTGLSPVYP..GEPSAPIRTAKAERRHQERLRMQSPELLAA  1671
Cattle Scribble                               ........................................EGLGPASPPPQGGGAPVRTAKAERRHQERLRVQSPELPAP  1462
Zebrafish Scribble                            SATPPSHCGSSGPSSIDGKGDSQRNSVEDSFRLEQRPNSMTGLIPVYP..GESAAPIRTAKAERRHQDRLRMQSPELTVA  1628
Coelacanth Scribble                           SCQLEKCQAKPKVSHVDGSGDSQRNSLEDGFRGDQRPSLMTGLSPVYPGSGDSSAPIRTAKAERRYQERLRMQSPELLTA  1211
Spotted gar Scribble                          PSVPLCVPR..LPQSGDRRGDSLRNSLEDDFRPEQRPNSMTGLSPVYP..SEPSAPIRTAKAERRHQERLRMQSPELLVG  1578
Chinese softshell turtle Scribble             .........................................RLIPVYPG.GEAGAPVRTAKAERRHQERLRMQSPEFLST  1396
Zebra finch Scribble                          ......................................SLGRLSP.....ADPGAPVRTAKAERRHQERLRMQSPELLGG  1411
Medaka Scribble                               SGSS.......GPSPVDSKGDSQRNSLEDSFRQEQRPNSMTGLIPAYS..GET.APIRTAKAERRHQERLRMQSPELAVA  1554
Yenyuan stream salamander Scribble isoform1   ...........GLSQVDGQGASHRNSLEDSLKSDHRPDSMTGLSPIYPGSGEPGAPIRTAKAERRHQDRVRMQSPELLTP   311
Yenyuan stream salamander Scribble isoform2   ...........GLSQVDGQGASHRNSLEDSLKSDHRPDSMTGLSPIYPGSGEPGAPIRTAKAERRHQDRVRMQSPELLTP   805
Yenyuan stream salamander Scribble isoform3   ...........GLSQVDGQGASHRNSLEDSLKSDHRPDSMTGLSPIYPGSGEPGAPIRTAKAERRHQDRVRMQSPELLTP   476
Yenyuan stream salamander Scribble isoform4   ...........GLSQVDGQGASHRNSLEDSLKSDHRPDSMTGLSPIYPGSGEPGAPIRTAKAERRHQDRVRMQSPELLTP   512
Yenyuan stream salamander Scribble isoform5   ...........GLSQVDGQGASHRNSLEDSLKSDHRPDSMTGLSPIYPGSGEPGAPIRTAKAERRHQDRVRMQSPELLTP   805
Yenyuan stream salamander Scribble isoform6   ...........GLSQVDGQGASHRNSLEDSLKSDHRPDSMTGLSPIYPGSGEPGAPIRTAKAERRHQDRVRMQSPELLTP   489
Yenyuan stream salamander Scribble isoform7   ...........GLSQVDGQGASHRNSLEDSLKSDHRPDSMTGLSPIYPGSGEPGAPIRTAKAERRHQDRVRMQSPELLTP   489
Yenyuan stream salamander Scribble isoform8   ...........GLSQVDGQGASHRNSLEDSLKSDHRPDSMTGLSPIYPGSGEPGAPIRTAKAERRHQDRVRMQSPELLTP   257
Yenyuan stream salamander Scribble isoform9   ...........GLSQVDGQGASHRNSLEDSLKSDHRPDSMTGLSPIYPGSGEPGAPIRTAKAERRHQDRVRMQSPELLTP   805

                                                        1680                1690                1700                1710                1720                1730                1740
conservation
Asian Arowana Scribble                        ASVDRELSPAERRALEAEKRAMWRAARPHPPTLSCTRPYGLEEDVRQYEKDLAKRLYQSRVRASQQLTAATSQATSQL..  1749
Cattle Scribble                               ...ERALSPAERRALEAEKRALWRAA......................................................  1485
Zebrafish Scribble                            L..DKELSPAEKRALEAEKRAMWRAA..........RPYGLEEDVRQYEQDLAKRLYQSRVRASLGSAAAPASSSCSSSS  1696
Coelacanth Scribble                           Q..DKDLSPAERRALEAEKRAMWRAA......................................................  1235
Spotted gar Scribble                          Q..DKELSPAEKRALEAEKRAMWRAA......................................................  1602
Chinese softshell turtle Scribble             Q..EKELSPAERRALEAEKRAMWRAA......................................................  1420
Zebra finch Scribble                          Q..DKELSPAERRALEAEKRAMWRAA......................................................  1435
Medaka Scribble                               P..DKDLSPAEKRALEAEKRAMWRAA......................................................  1578
Yenyuan stream salamander Scribble isoform1   Q..ETDLSPAERRALEAEKRAMWRAA............STLEESIKQYEQDLARKLYQSRQRVPAPGQTSSPAQTPIPGA   377
Yenyuan stream salamander Scribble isoform2   Q..ETDLSPAERRALEAEKRAMWRAA............STLEESIKQYEQDLARKLYQSRQRVPAPGQTSSPAQTPIPGA   871
Yenyuan stream salamander Scribble isoform3   Q..ETDLSPAERRALEAEKRAMWRAA......................................................   500
Yenyuan stream salamander Scribble isoform4   Q..ETDLSPAERRALEAEKRAMWRAA......................................................   536
Yenyuan stream salamander Scribble isoform5   Q..ETDLSPAERRALEAEKRAMWRAA......................................................   829
Yenyuan stream salamander Scribble isoform6   Q..ETDLSPAERRALEAEKRAMWRAA......................................................   513
Yenyuan stream salamander Scribble isoform7   Q..ETDLSPAERRALEAEKRAMWRAA............STLEESIKQYEQDLARKLYQSRQRVPAPGQTSSPAQTPIPGA   555
Yenyuan stream salamander Scribble isoform8   Q..ETDLSPAERRALEAEKRAMWRAA......................................................   281
Yenyuan stream salamander Scribble isoform9   Q..ETDLSPAERRALEAEKRAMWRAA......................................................   829

                                                      1750                1760                1770                1780                1790                1800                1810                1820
conservation
Asian Arowana Scribble                        .......RMKSLEQDALKAQMVIAKSQDMK.KRGTLDRLTESPSPAPTPSPTPLEDTSPRTVTSPGRLSLSEKKFDYRQF  1821
Cattle Scribble                               .......RMKSLEQDALRAQMVLSKSQDGRSKRGPLERLAEAPSPAPTPSPTPLEDLGPQTGTSPGRL............  1546
Zebrafish Scribble                            SCQP...RMKSLEQDALKAQMVIAKSKEGK.KRGTLDQLSESPSPAPTPSPTPMEDISPRTVTSPGRLSLSEKKFDYRQF  1772
Coelacanth Scribble                           .......RMKSLEQDALKAQMVIAKSKDGK.KRSTLEQLAESPSPAPTPSPTPLEDYSPRAVTSPGRL............  1295
Spotted gar Scribble                          .......RMKSLEQDALKAQMVIAKSKEGK.KRNTLDQLAESPSPAPTPSPTPLEDYSPRSVTSPGRL............  1662
Chinese softshell turtle Scribble             .......RMKSLEQDALKAQMVIAKSKEGK.KRSTLEQLAESPSPAPTPSPT............................  1464
Zebra finch Scribble                          .......RMKSLEQDALKAQMVIAKSKEGK.KRSTLEQLAESPSPAPTPSPTPLEDCSPRNVTSPGRL............  1495
Medaka Scribble                               .......RMKSLEQDALKAQMVIAKSRDGK.KRGTLDQLTESPSPAPTPSPTPMEELSPRGLTSPGRL............  1638
Yenyuan stream salamander Scribble isoform1   AGSAGHSRMKSLEQDALKAQMVIAKSKEGR.KRSTLDQLAESPSPAPTPSPTPLEDYSPRNVTSPGRLSISEKKFDYREF   456
Yenyuan stream salamander Scribble isoform2   AGSAGHSRMKSLEQDALKAQMVIAKSKEGR.KRSTLDQLAESPSPAPTPSPTPLEDYSPRNVTSPGRLSISEKKFDYREF   950
Yenyuan stream salamander Scribble isoform3   .......RMKSLEQDALKAQMVIAKSKEGR.KRSTLDQLAESPSPAPTPSPTPLE.........................   547
Yenyuan stream salamander Scribble isoform4   .......RMKSLEQDALKAQMVIAKSKEGR.KRSTLDQLAESPSPAPTPSPTPLE.........................   583
Yenyuan stream salamander Scribble isoform5   .......RMKSLEQDALKAQMVIAKSKEGR.KRSTLDQLAESPSPAPTPSPTPLE.........................   876
Yenyuan stream salamander Scribble isoform6   .......RMKSLEQDALKAQMVIAKSKEGR.KRSTLDQLAESPSPAPTPSPTPLE.........................   560
Yenyuan stream salamander Scribble isoform7   AGSAGHSRMKSLEQDALKAQMVIAKSKEGR.KRSTLDQLAESPSPAPTPSPTPLE.........................   609
Yenyuan stream salamander Scribble isoform8   .......RMKSLEQDALKAQMVIAKSKEGR.KRSTLDQLAESPSPAPTPSPTPLEDYSPRNVTSPGRLSISEKKFDYREF   353
Yenyuan stream salamander Scribble isoform9   .......RMKSLEQDALKAQMVIAKSKEGR.KRSTLDQLAESPSPAPTPSPTPLEDYSPRNVTSPGRL............   889
```

```
                                                    1830      1840      1850      1860           1870      1880      1890
conservation
Asian Arowana Scribble                         AAIPSSKPVYDIQSPDNADDLRYMDSSGNCGAPEEPDIEVAL.....PLPATSALEEMALYSSKRKLRQGRRSLEAAVPT  1896
Cattle Scribble                                .............SPDFAEELRSLEPPPSPGLPEE.DGEVAMVLLGRPSPSSTGPEEVALCSSRRAVRPGRRG.LGPVPS  1611
Zebrafish Scribble                             AAIPSSKPVYDIQSPGAADDVHFMDESSSN............................................AGDFLG  1808
Coelacanth Scribble                            .............VHGHSDSVVLLENESCLAV....TVFTEIFGPSNQILTTPVFRVSDRYSNLEKKKSPEKSIESPAPK  1358
Spotted gar Scribble                           .............SPDISGDIHFIDDNSNSPAPP....VDTEAEVHIPLPTTSAIEEMALYSNRRKLRHGRRSLETAVPT  1725
Chinese softshell turtle Scribble              ................................................................................  1464
Zebra finch Scribble                           ................................................................................  1495
Medaka Scribble                                .............TPDTIDDMKFIDD....GSSSQGRAASPEAEMQTAPPATSALEEMALYSNKRKLRQGRRSLETAVPT  1701
Yenyuan stream salamander Scribble isoform1    AAIPSSKPVYEIQSPDFGHD............................................................   476
Yenyuan stream salamander Scribble isoform2    AAIPSSKPVYEIQSPDFGHDLKYMDDTKVSARPDDGEVEDADLAENIAVPNPSALEEMSVYSNKRKLRHTRRSLETAIPT  1030
Yenyuan stream salamander Scribble isoform3    ..........................................................................VSRLWT   553
Yenyuan stream salamander Scribble isoform4    ..........................................................................VSRLWT   589
Yenyuan stream salamander Scribble isoform5    ..........................................................................VSRLWT   882
Yenyuan stream salamander Scribble isoform6    ..........................................................................VSRLWT   566
Yenyuan stream salamander Scribble isoform7    ..........................................................................VSRLWT   615
Yenyuan stream salamander Scribble isoform8    AAIPSSKPVYEIQSPDFGHD............................................................   373
Yenyuan stream salamander Scribble isoform9    .............SPDFGHDLKYMDDTKVSARPDDGEVEDADLAENIAVPNPSALEEMSVYSNKRKLRHTRRSLETAIPT   956
```

X non conserved

X $\geq$60% conserved

X $\geq$85% conserved
