# Supplementary material for: The First Transcriptome Assembly of Yenyuan Stream Salamander (Batrachuperus yenyuanensis) Provides Novel Insights into Its Molecular Evolution
Source: Int J Mol Sci. 2019 Mar 27;20(7):1529. doi: 10.3390/ijms20071529 (PMC6480382; doi:10.3390/ijms20071529)
Supplement: Supplementary file 1 [file ijms-20-01529-s001.zip › Supplenmental Tables.docx]

Supplementary Table 1. The completeness estimation of predicted protein-coding sequences from transcriptome of Yenyuan stream salamanders by BUSCOs

| Category | BUSCOs hits | % of Genome |
| --- | --- | --- |
| Complete BUSCOs (C) | 1,585 | 40.1 % |
| Complete and single-copy BUSCOs (S) | 1,299 | 32.9 % |
| Complete and duplicated BUSCOs (D) | 286 | 7.2 % |
| Fragmented BUSCOs (F) | 330 | 8.4 % |
| Missing BUSCOs (M) | 2,035 | 51.5 % |
| Total BUSCO groups searched | 3,950 |  |

Supplementary Table 2. The annotated state of all predicted encoding sequence based on Nr, Swissprot, KEGG and KOG.

| Database | Nr | Swissprot | KEGG | KOG | Overall |
| --- | --- | --- | --- | --- | --- |
| Number | 12,392 | 11,190 | 10,615 | 9,393 | 12,462 |
| Percentage | 88.29% | 79.73% | 75.63% | 66.93% | 88.79% |

Supplementary Table 3: The functional annotation of the top 20 expression of gene

| Expression | Protein | FPKM | Full name | Function |
| --- | --- | --- | --- | --- |
| 1 | EEF1A1 | 15004.03 | Elongation factor 1-alpha | protein synthesis |
| 2 | Unknown | 12220.73 | NA | NA |
| 3 | MYLPF | 10191.5 | Myosin regulatory light chain 2, skeletal muscle isoform type 2 | This chain binds calcium. |
| 4 | ACTA2 | 9785.69 | Actin, aortic smooth muscle | Actins are highly conserved proteins that are involved in various types of cell motility and are ubiquitously expressed in all eukaryotic cells. |
| 5 | FTH1 | 8137.79 | Ferritin heavy chain B | Stores iron in a soluble, non-toxic, readily available form. Important for iron homeostasis. Has ferroxidase activity. Iron is taken up in the ferrous form and deposited as ferric hydroxides after oxidation |
| 6 | CKM | 7984.02 | Creatine kinase M-type | Reversibly catalyzes the transfer of phosphate between ATP and various phosphogens (e.g. creatine phosphate). Creatine kinase isoenzymes play a central role in energy transduction in tissues with large, fluctuating energy demands, such as skeletal muscle, heart, brain and spermatozoa. |
| 7 | Unknown | 7582.42 | NA | NA |
| 8 | RPS6 | 7334.68 | 40S ribosomal protein S6 | Ribosomes, the organelles that catalyze protein synthesis, consist of a small 40S subunit and a large 60S subunit. |
| 9 | APOC1 | 6142.25 | Apolipoprotein C-I | The protein encoded by this gene is a member of the apolipoprotein C family. This gene is expressed primarily in the liver, and it is activated when monocytes differentiate into macrophages. |
| 10 | Hbb-b1 | 6141.04 | Hemoglobin subunit beta-1 | Involved in oxygen transport from the lung to the various peripheral tissues. |
| 11 | Parvalbumin beta | 5944.76 | Parvalbumin beta | In muscle, parvalbumin is thought to be involved in relaxation after contraction. It binds two calcium ions (By similarity). |
| 12 | AVD | 5877.48 | Avidin | Avidin is a tetrameric biotin-binding protein produced in the oviducts of birds, reptiles and amphibians and deposited in the whites of their eggs. Dimeric members of the avidin family are also found in some bacteria. In chicken egg white, avidin makes up approximately 0.05% of total protein (approximately 180 μg per egg). The tetrameric protein contains four identical subunits (homotetramer), each of which can bind to biotin (Vitamin B7, vitamin H) with a high degree of affinity and specificity. |
| 13 | MYL1 | 5376.12 | Myosin light chain 1/3, skeletal muscle | Regulatory light chain of myosin. Does not bind calcium. |
| 14 | HBA2 | 5261.31 | Hemoglobin subunit alpha-2 | hemoglobin |
| 15 | RPL27A | 4738.98 | 60S ribosomal protein L27a | Ribosomes, the organelles that catalyze protein synthesis, consist of a small 40S subunit and a large 60S subunit. |
| 16 | ENO3 | 4230.44 | Beta-enolase | Appears to have a function in striated muscle development and regeneration. |
| 17 | EEF2 | 3809.17 | Elongation factor 2 | protein synthesis |
| 18 | FABP7 | 3607.84 | Fatty acid-binding protein, brain | B-FABP could be involved in the transport of a so far unknown hydrophobic ligand with potential morphogenic activity during CNS development. It is required for the establishment of the radial glial fiber system in developing brain, a system that is necessary for the migration of immature neurons to establish cortical layers |
| 19 | RPL4 | 3583.66 | 60S ribosomal protein L4 | Ribosomes, the organelles that catalyze protein synthesis, consist of a small 40S subunit and a large 60S subunit. |
| 20 | HSP | 3448.11 | Heat shock protein | Many members of this group perform chaperone function by stabilizing new proteins to ensure correct folding or by helping to refold proteins that were damaged by the cell stress. |

Supplementary Table 4: The result of gene families clustering from Yenyuan stream salamander and other 18 vertebrates

| Species | Genes number | Genes in families | Unclustered genes | Family number | Unique families | Average genes per family |
| --- | --- | --- | --- | --- | --- | --- |
| zebrafish | 25709 | 23916 | 1793 | 13937 | 221 | 1.72 |
| coelacanth | 19568 | 18571 | 997 | 13172 | 131 | 1.41 |
| spotted gar | 18341 | 17391 | 950 | 13437 | 57 | 1.29 |
| fugu | 19682 | 17985 | 1697 | 12458 | 145 | 1.44 |
| Chinese softshell turtle | 18177 | 17107 | 1070 | 12431 | 76 | 1.38 |
| zebra finch | 17472 | 15361 | 2111 | 11527 | 70 | 1.33 |
| medaka | 18518 | 17678 | 840 | 12124 | 48 | 1.46 |
| cattle | 19981 | 18965 | 1016 | 14291 | 139 | 1.33 |
| elephant shark | 19491 | 16099 | 3392 | 12631 | 100 | 1.27 |
| whale shark | 23554 | 20232 | 3322 | 13424 | 195 | 1.51 |
| red junglefowl | 18342 | 17107 | 1235 | 12376 | 136 | 1.38 |
| human | 19696 | 18773 | 923 | 14385 | 129 | 1.31 |
| sea lamprey | 10202 | 7919 | 2283 | 6373 | 89 | 1.24 |
| Asian arowana | 22016 | 18563 | 3453 | 12698 | 33 | 1.46 |
| African clawed frog | 33574 | 26048 | 7526 | 16299 | 408 | 1.6 |
| Western clawed frog | 45942 | 36657 | 9285 | 16985 | 939 | 2.16 |
| American bullfrog | 22214 | 18525 | 3689 | 8466 | 366 | 2.19 |
| high Himalaya frog | 24745 | 23480 | 1265 | 13544 | 104 | 1.73 |
| Yenyuan stream salamander | 14035 | 10829 | 3206 | 7088 | 278 | 1.53 |
