# Supplementary figures and images for: The First Transcriptome Assembly of Yenyuan Stream Salamander (Batrachuperus yenyuanensis) Provides Novel Insights into Its Molecular Evolution
Source: Int J Mol Sci. 2019 Mar 27;20(7):1529. doi: 10.3390/ijms20071529 (PMC6480382; doi:10.3390/ijms20071529)

Length distributions of raw contigs assembled by Trinity

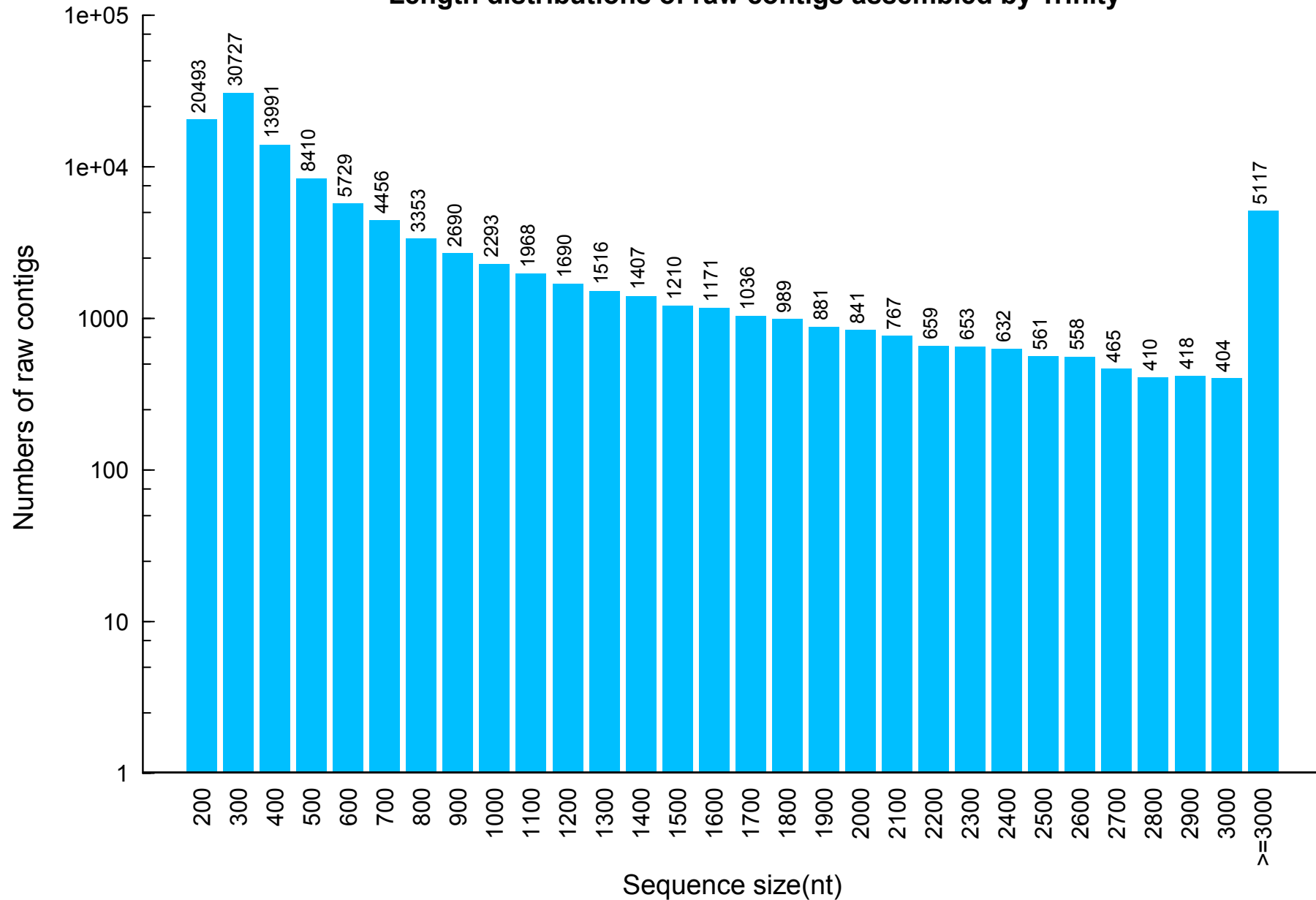

Supplement: Supplementary file 1 [file ijms-20-01529-s001.zip › Figure S1.pdf]

Length distribution of Unigenes

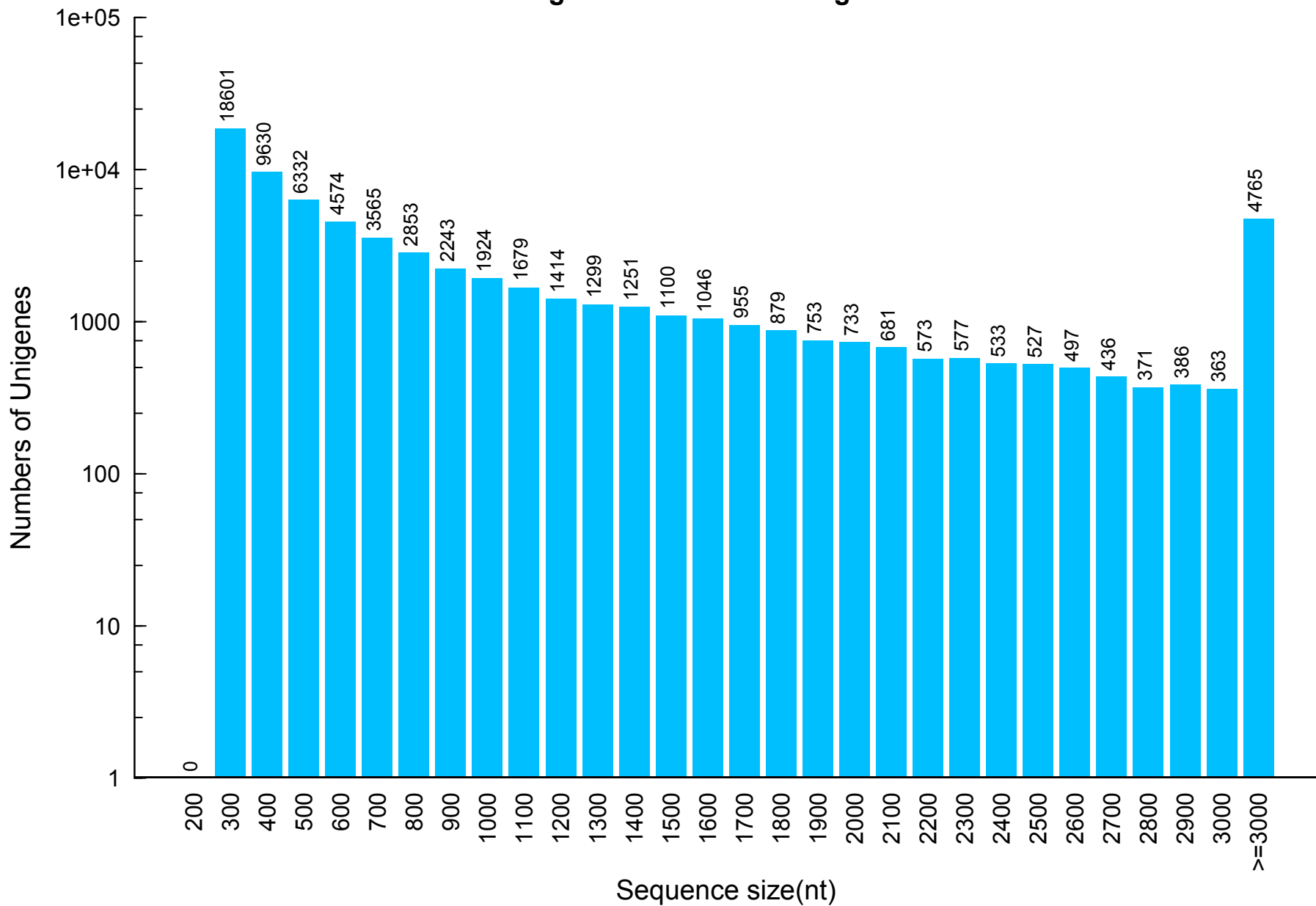

Supplement: Supplementary file 1 [file ijms-20-01529-s001.zip › Figure S2.pdf]

Length distribution of unigenes with FPKM  $\geq 1$

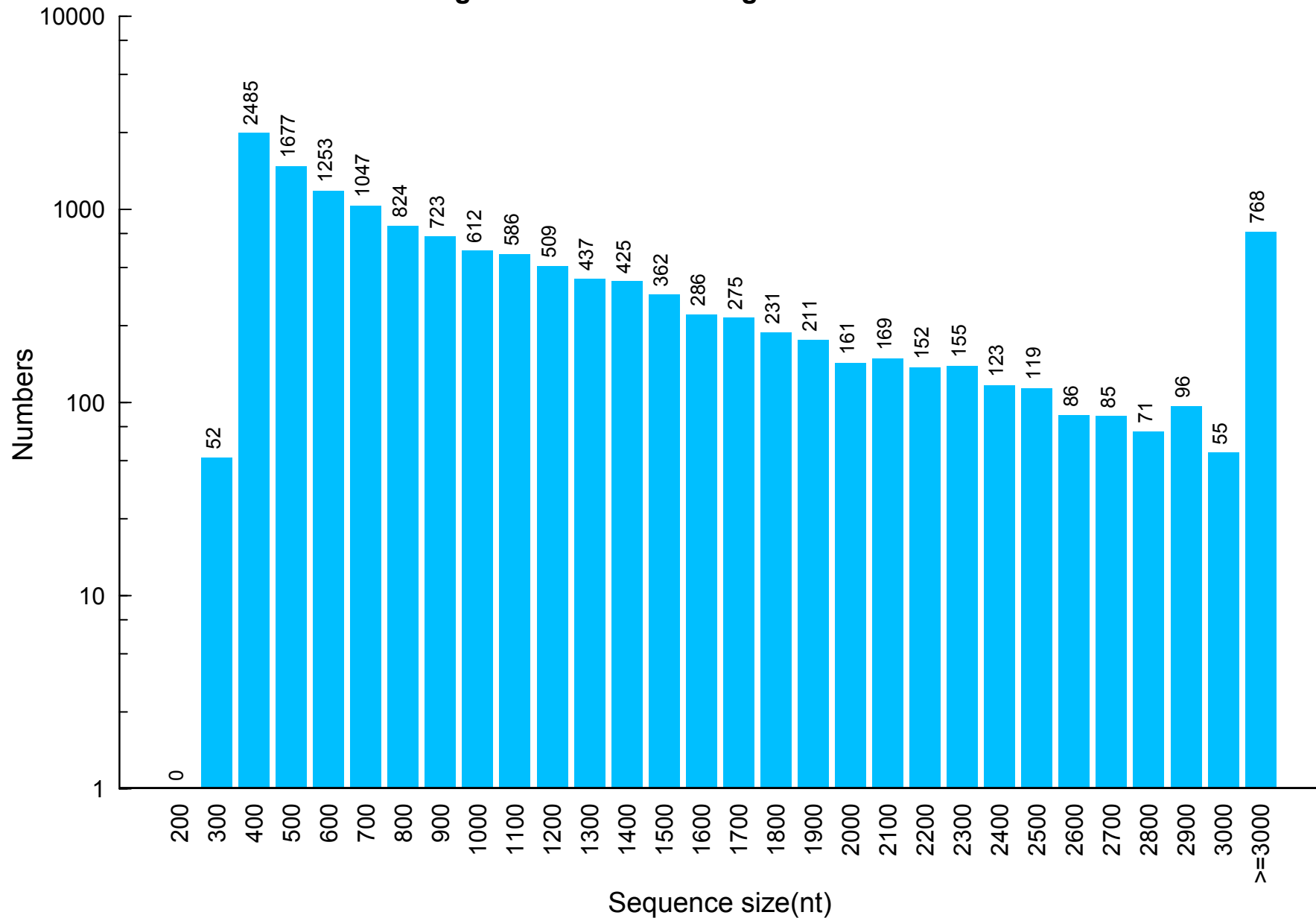

Supplement: Supplementary file 1 [file ijms-20-01529-s001.zip › Figure S3.pdf]
